# Cancer cells suppress NK cell activity by actin-driven polarization of inhibitory ligands to the immunological synapse

**DOI:** 10.1073/pnas.2503259122

**Published:** 2025-08-05

**Authors:** Céline Hoffmann, Liza Filali, Hannah Wurzer, Diogo Pereira Fernandes, Takouhie Mgrditchian, Wanxin Huang, Flora Moreau, Max Krecké, Clément Thomas

**Affiliations:** ^a^Cytoskeleton and Cancer Progression, Department of Cancer Research, Luxembourg Institute of Health, Luxembourg City L-1210, Luxembourg; ^b^National Cytometry Platform, Luxembourg Translational Medicine Operations Hub, Luxembourg Institute of Health, Esch-sur-Alzette L-4354, Luxembourg; ^c^Faculty of Science, Technology and Medicine, Doctoral School in Science and Engineering, University of Luxembourg, Esch-sur-Alzette L-4364, Luxembourg

**Keywords:** immunological synapse, cancer, natural killer, actin cytoskeleton

## Abstract

Natural killer (NK) cells engage target cells via the immunological synapse (IS), where inhibitory and activating signals determine whether NK cell cytotoxicity is suppressed or activated. We previously reported that cancer cells can rapidly remodel their actin cytoskeleton upon NK cell engagement, leading to F-actin accumulation at the synapse. Here, we show that this process inhibits NK cell activation as indicated by impaired MTOC and lytic granule polarization. Exploring the underlying mechanism, we demonstrate that actin remodeling drives the recruitment of inhibitory ligands, such as HLA-A, -B, and -C, to the synapse. Disrupting HLA interaction with their cognate inhibitory receptors KIRs restores NK cell activation. Using NK cells expressing inhibitory KIR receptors, matched or unmatched to HLA molecules on cancer cells, we show that synaptic F-actin accumulation and matching KIR–HLA interactions jointly suppress NK cell cytotoxicity. Our findings reveal an immune evasion strategy in which cancer cells impair NK cell activation by altering synaptic signaling through actin cytoskeleton–driven recruitment of inhibitory signals to the IS.

## Significance

Our findings provide insights into previously uncharacterized dynamics at the cancer cell–cytotoxic lymphocyte interface, uncovering a critical mechanism by which actin cytoskeleton remodeling at the cancer cell side promotes resistance to natural killer (NK) cell–mediated cytotoxicity. We demonstrate that actin-driven polarization of NK cell inhibitory ligands to the cancer cell side of the immunological synapse amplifies inhibitory signaling, thereby suppressing NK cell lytic activity. These findings shed light on previously uncharacterized molecular strategies employed by cancer cells to evade NK cell–mediated cytotoxicity and may have important implications for advancing our understanding of cancer immune evasion and developing more effective immunotherapeutic interventions.

Natural killer (NK) cells are cytotoxic lymphocytes that play a crucial role in cancer immunosurveillance. Unlike CD8+ T cells, which require prior sensitization to specific antigens and clonal expansion, NK cells can recognize and eliminate abnormal cells upon first encounter, positioning them as a critical first line of defense against tumorigenesis. Instead of antigen-specific receptors, NK cells express a repertoire of germline-encoded inhibitory and activating receptors that collectively guide their response (1). Activating receptors detect stress-induced or abnormal ligands on target cells, pushing NK cells toward cytotoxic activation, while inhibitory receptors recognize self-molecules signaling cellular normalcy and restraining NK cell activation (1, 2). The decision of NK cells to kill or spare potential targets is ultimately determined by the balance of inhibitory and activating signals received through these receptors. This finely tuned interplay enables NK cells to mount a cytotoxic response against the diverse phenotypes exhibited by cancer cells while preventing inappropriate cytotoxic responses against healthy cells. Although NK cells are primarily associated with the innate immune system, they can also display adaptive properties which may be harnessed for cancer immunotherapy (1, 3).

Healthy cells typically express high levels of major histocompatibility complex class I (MHC I) molecules on their surface, which serve as “self” markers. These molecules play a critical role in promoting NK cell tolerance by engaging the main inhibitory receptors, including inhibitory killer-cell immunoglobulin-like receptors (iKIRs) and the CD94/NKG2A heterodimeric receptor (4–6). In contrast, tumors frequently downregulate MHC-I molecules on their surface, reducing detection by CD8+ T cells, which require MHC-I to present cancer cell antigens for immune recognition. This reduction in self-signals (or “missing-self”) weakens the inhibitory input mediated by iKIRs and NKG2A, making NK cells more prone to activation (7). However, NK cell activation also requires additional inputs from activating receptors, such as NKG2D and the natural cytotoxicity receptors (NCRs), including NKp30, NKp44, and NKp46 (8). For instance, stress molecules like MICA, MICB, and UL16-binding proteins (ULBPs) are frequently overexpressed on tumor cells, serving as potent activating ligands for NKG2D. Additionally, the stress-induced self-molecule B7-H6 is upregulated in various cancers and specifically engages NKp30, leading to robust NK cell activation (9). When an activating receptor binds to its ligand, intracellular signaling is initiated through distinct motifs in adaptor molecules (6). Immunoreceptor tyrosine-based activation motif (ITAM)-bearing adaptors, such as DAP12, rely on phosphorylation by Src family kinases to create docking sites for downstream signaling kinases, such as Syk and Zap70. In contrast, ITAM-independent activating receptors like NKG2D signal through adaptors, such as DAP10 which contains a YINM motif (10). Phosphorylation of this motif recruits PI3K and other signaling proteins. While both DAP12 and DAP10 use different signaling intermediates, both pathways converge on actin cytoskeleton polymerization and reorganization (11, 12). Conversely, engagement of inhibitory receptors with their ligands leads to phosphorylation of immunoreceptor tyrosine-based inhibition motifs (ITIMs) within their cytoplasmic domain by Src family kinases (6). Phosphorylated ITIMs recruit of phosphatases, particularly Src homology 2-domain-containing protein tyrosine phosphatase-1 (SHP-1) and SHP-2, which dephosphorylate key molecules in the activation pathway, thereby suppressing the activation cascade (6, 13, 14). In addition, an increasing number of studies support that NK cells are also regulated by non-MHC-I-specific immune checkpoints, such as PD-1, LAG-3, TIM-3, and TIGIT, which have been reported to induce NK cell functional exhaustion (15–18). However, the consistency and prevalence of these pathways in directly modulating NK cell anti-tumor activity across various human cancers are still under evaluation.

The recognition of cancer cells and their subsequent killing by NK cells rely on the formation a stable cell-to-cell contact known as the lytic immunological synapse (IS). This process initiates with NK cell adhesion to the target cell, typically mediated by the interaction between lymphocyte function-associated antigen-1 (LFA-1) on NK cells and intracellular adhesion molecules (ICAMs) on the cancer cell surface (19). This adhesion is strengthened by the engagement of activating receptors, leading to the reorganization of the actin cytoskeleton into a circular network of filaments at the periphery of the IS, and the polarization of the microtubule organizing center (MTOC) and associated cytotoxic granules toward the target cell (19–22). In addition to releasing classical cytotoxic granules containing perforins, granzymes, and other cytotoxic molecules, NK cells have recently been shown to secrete membraneless particles termed supramolecular attack particles (SMAPs) containing perforin and granzyme B surrounded by a thrombospondin-1 shell (23). The actin cytoskeleton plays multiple and critical roles in NK cell–mediated cytotoxicity, supporting IS initiation, stabilization, and maturation into distinct functional domains (24, 25). During degranulation, actin dynamics regulate the formation of localized clearances within the synaptic cortical F-actin meshwork, allowing the granules to pass through and fuse with the cell membrane (26–30). Additionally, actin dynamics contribute to the regulation of synaptic signaling through various mechanisms, including the generation of forces that modulate receptor–ligand interactions, the regulation of the conformation and activity of critical checkpoint molecules such as SHP-1, and the assembly and transport of signaling clusters to specific regions of the IS (12, 31, 32).

While the actin cytoskeleton is widely recognized as a critical and multifunctional component of ISs formed by NK cells and CD8+ T cells with their targets, its role(s) within the attacked cancer cells remain comparatively understudied (24). Nevertheless, growing evidence indicates that the configuration and dynamic remodeling of the actin cytoskeleton in cancer cells during interactions with cytotoxic lymphocytes critically influence IS outcomes (24, 33). The cortical actin cytoskeleton of cancer cells—a key regulator of their mechanical properties—plays a pivotal role in generating synaptic forces that modulates the activation of mechanosensitive immunoreceptors and the pore-forming activity of perforin (34–38). In addition, our previous work established that accumulation of F-actin at the cancer cell side of the IS during interactions with NK cells triggers resistance to NK cell–mediated cytotoxicity (39, 40). This synaptic evasion strategy is conserved across multiple breast cancer and chronic lymphocytic leukemia (CLL) cell lines and has been validated in primary CLL patient samples. However, the molecular mechanism by which actin cytoskeleton remodeling protects cancer cells during NK cell attack remains unknown.

## Results

### Synaptic Polarization of the Cancer Cell Actin Cytoskeleton Inhibits MTOC and Cytotoxic Granule Polarization in Primary NK Cells.

**Fig. 1.**
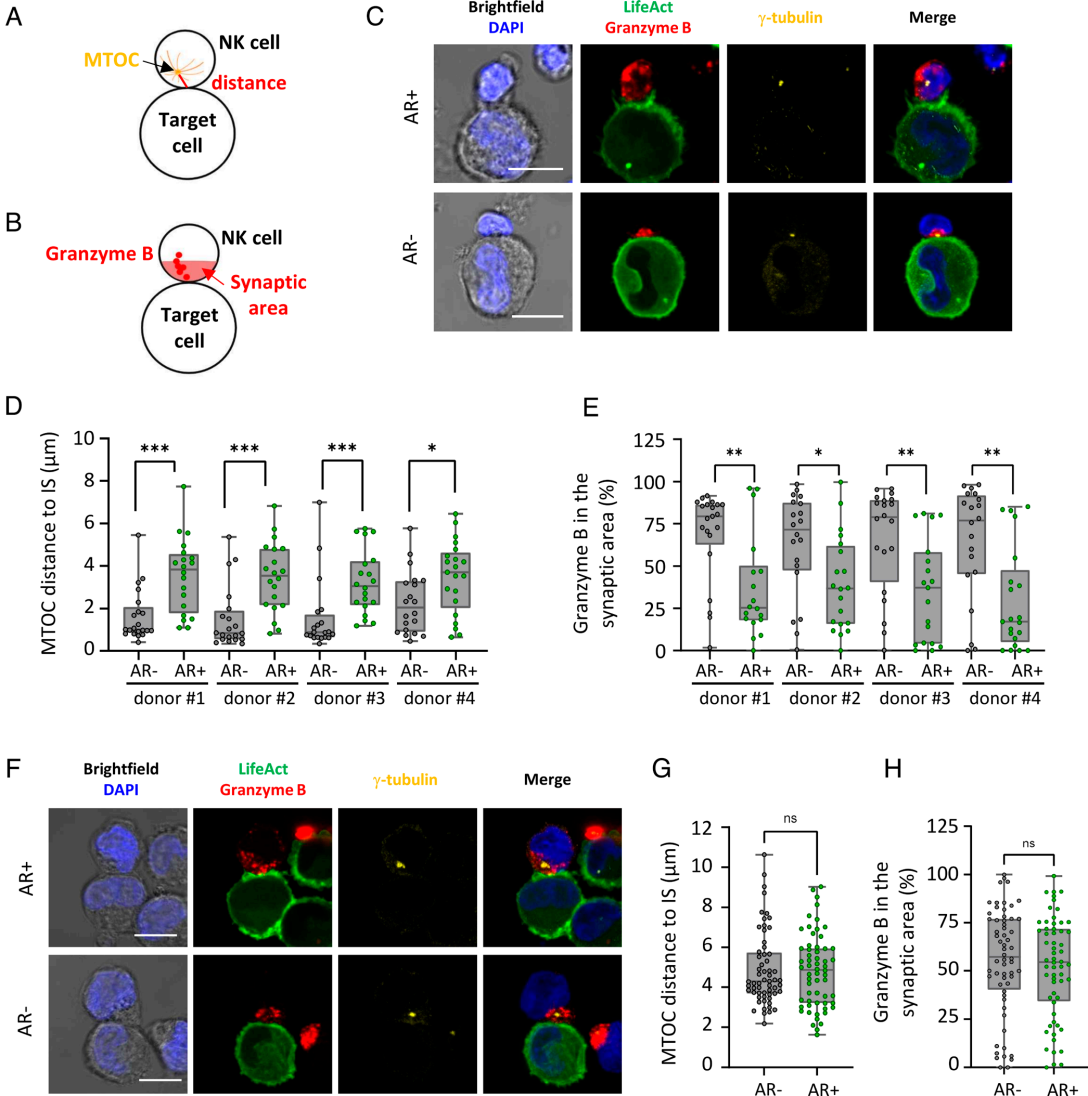
F-actin polarization at the cancer cell side of the IS is associated with impaired lytic machinery polarization in primary NK cells. Emerald-LifeAct-expressing MDA-MB-231 cells (green) were cocultured with NK cells for 60 min, followed by immunolabeling for Granzyme B, γ-Tubulin along with DAPI staining for the nucleus. NK cell lytic machinery polarization was assessed by measuring the distance between the MTOC and the IS center (*A*), and by quantifying the proportion of Granzyme B localized within the synaptic area, defined as the proximal third of the cell closest to the IS (*B*). (*C*) Representative confocal images (maximum intensity projection of four optical slices) of cell-to-cell conjugates between primary NK cells and MDA-MB-231 cells, with or without synaptic actin cytoskeleton remodeling (AR+ and AR−, respectively). (*D* and *E*) Quantitative analysis of primary NK cell lytic machinery polarization, collected from NK cells isolated from four distinct donors, with n = 20 cell-to-cell conjugates analyzed per condition. Data on the MTOC distance from the IS (*D*) and Granzyme B enrichment at the IS (*E*) are presented. (*F*) Representative confocal images of cell-to-cell conjugates between NK-92MI cells and AR+ or AR− MDA-MB-231 cells. (*G* and *H*) Quantitative analysis of NK-92MI cell lytic machinery polarization, collected from three independent experiments, with n = 60 cell-to-cell conjugates analyzed per condition. Data on the MTOC distance from the IS (*G*) and Granzyme B enrichment at the IS (*H*) are presented. Statistical significance was determined using the Mann–Whitney test. (Scale bars, 10 µm.)

We previously established that the rapid and sustained polarization of F-actin at the IS in cancer cells during interactions with NK cells significantly enhances their resistance to NK cell–mediated cytotoxicity (39, 40). Notably, cancer cells undergoing actin cytoskeleton remodeling exhibited reduced levels of NK cell–delivered granzyme B, suggesting a potential impairment of NK cell cytotoxic activation. To investigate this hypothesis, we assessed NK cell activation status during interactions with MDA-MB-231 breast cancer cells, comparing targets with or without F-actin synaptic polarization. Primary NK cells were isolated from healthy donors and used as effector cells. Live cell imaging analyses confirmed that primary NK cells induce a rapid and sustained cytoskeletal response in a subset of MDA-MB-231 target cells (*SI Appendix*, Fig. S1 *A*–*C* and Movies S1–S3 for actin remodeling-positive cells; *SI Appendix*, Fig. S1 *E*–*G* and Movies S5–S7 for remodeling-negative cells). In remodeling-positive cells, F-actin accumulation at the IS typically began shortly after NK cell contact and persisted until detachment. Conjugate duration varied widely, from approximately 5 min to over 2 h. In contrast, remodeling-negative cells exhibited early signs of death, most notably membrane blebbing, within 20 min of conjugate formation. The protective effect of actin remodeling was further supported by imaging flow cytometry (IFC), which showed significantly lower rates of apoptosis and necrosis in remodeling-positive cells (*SI Appendix*, Fig. S2). The activation of effector cells was evaluated by visualizing the microtubule-organizing center (MTOC) via γ-tubulin staining and measuring its distance from the center of the IS (Fig. 1 *A* and *C*). Additionally, cytotoxic granules were detected via granzyme B staining, and their polarization was quantified as the percentage of granzyme B localized within the third of the NK cell closest to the IS, or “synaptic area” (Fig. 1 *B* and *C*). For each donor-specific NK cell preparation, 40 cell–cell conjugates were analyzed via confocal microscopy (20 for each target cell actin phenotype). Our data reveal a statistically significant and striking difference in NK cell polarization depending on the F-actin configuration in target cells. NK cells interacting with target cells exhibiting synaptic F-actin accumulation showed a marked reduction in MTOC and granule polarization compared to those engaging with target cells lacking actin cytoskeleton remodeling (Fig. 1 *C*–*E*).

To assess whether the NK cell polarization defect is dependent on iKIR signaling, we extended our analysis using NK-92MI cells, an IL-2-independent variant of the NK-92 cell line that lacks iKIRs and is therefore unresponsiveness to MHC-I (41). Unlike primary NK cells, these effector cells displayed a similarly pronounced polarization of both their MTOC and granules toward target cells, irrespective of the target cell’s actin phenotype (n = 60; *P* = ns; Fig. 1 *F*−*H*). Taken together, these findings underscore the pivotal role of actin cytoskeleton remodeling in determining whether MDA-MB-231 cells adopt an inhibitory or stimulatory phenotype toward primary NK cells and suggest that this outcome depends on inhibitory signaling, particularly involving iKIRs.

### Synaptic Polarization of the Cancer Cell Actin Cytoskeleton Correlates with Localized Enrichment of Surface HLA Inhibitory Ligands.

**Fig. 2.**
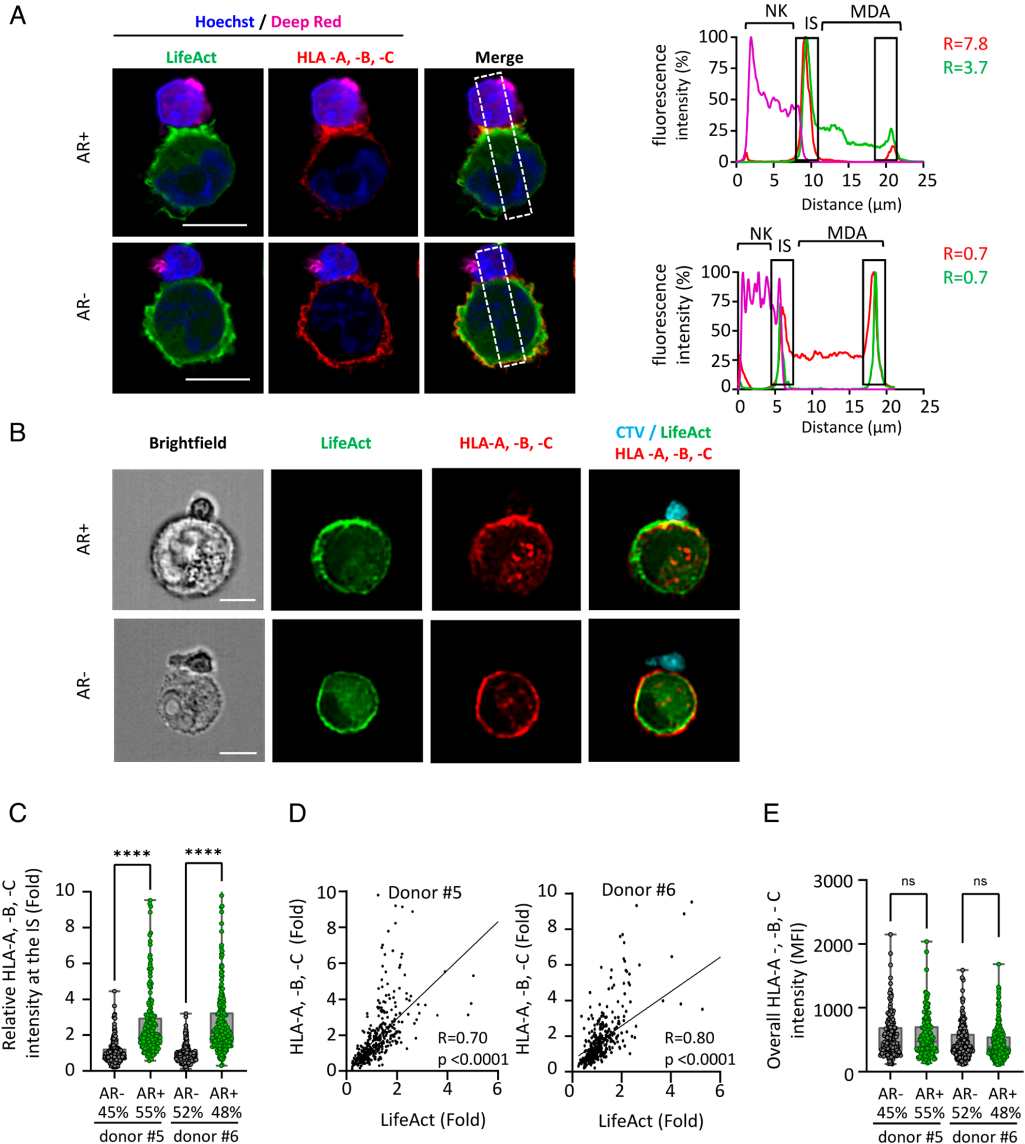
Polarization of the actin cytoskeleton at the cancer cell side of the IS correlates with the local accumulation of HLA molecules during interaction with NK cells. (*A*) Emerald-LifeAct-expressing MDA-MB-231 cells (green) were prelabeled for HLA-A, -B, and -C (red) and cocultured for 40 min with Deep Red–stained primary NK cells (magenta), along with Hoechst staining for the nucleus (blue). Representative Airyscan images show cell-to-cell conjugates formed between primary NK cells and MDA-MB-231 cells with or without synaptic actin cytoskeleton remodeling (AR+ and AR−, respectively). The dashed rectangle indicates the 50-pixel-wide line used to measure mean fluorescence intensity (MFI) for Emerald-LifeAct, HLA-A, -B, and -C, and Deep Red signals. The upper chart shows MFI profiles for AR+ MDA-MB-231 cells, while the lower chart displays profiles for AR- MDA-MB-231 cells. The relative intensity of HLA-A, -B, and -C (red), Emerald-LifeAct (green), and Deep Red (magenta) at the IS is displayed on the plot. (*B*–*E*) Emerald-LifeAct-expressing MDA-MB-231 cells (green) were prelabeled for HLA-A, -B, and -C (red) and cocultured for 40 min with CTV-stained primary NK cells (cyan). Data were collected from NK cells isolated from three distinct donors, with n = 200 cell-to-cell conjugates analyzed per condition. (*B*) Representative IFC images of cell-to-cell conjugates between primary NK cells and AR+ or AR− MDA-MB-231 cells. The relative Emerald-LifeAct intensity at the IS was used to classify conjugates into AR+ and AR− groups (ratio >1 and ratio <1, respectively). (*C*) Relative HLA-A, -B, and -C intensities at the IS are shown for these two subgroups. Statistical significance was determined using the Mann–Whitney test. The percentage of conjugates of each subgroup is indicated for each condition. (*D*) Correlation graph showing the relative intensities of Emerald-LifeAct and HLA-A, -B, and -C at the IS across the entire population of cell-to-cell conjugates analyzed in (*C* and *D*), without distinguishing between AR+ or AR− MDA-MB-231 cells. The correlation was determined using Spearman’s correlation coefficient. (*E*) The overall MFI of HLA-A, -B, and -C across the entire cell membrane of the cancer cell was measured in cell-to-cell conjugates formed between primary NK cells and AR+ or AR− MDA-MB-231 cells. The percentage of conjugates of each subgroup is indicated for each condition. Statistical significance was determined using the Mann–Whitney test. (Scale bars, 10 µm.)

Building on the above findings, we investigated the distribution of classical MHC-I molecules (HLA-A, -B, and -C), the main inhibitory ligands for NK cells, on the surface of target cells conjugated with primary NK cells. High-resolution confocal microscopy revealed a marked accumulation of HLA-A, -B, and -C molecules at the IS in target cells exhibiting synaptic actin cytoskeleton polarization (Fig. 2*A*). This synaptic enrichment resulted in a several-fold increase in fluorescence intensity at the IS compared to the nonsynaptic regions of the same cell. Conversely, target cells lacking synaptic F-actin polarization displayed a relatively uniform distribution of HLA-A, -B, and -C molecules, with occasional reductions in signal intensity at the IS. To increase the statistical power of the analysis, IFC was employed to examine a large cohort of NK cell–target cell conjugates using primary NK cells from two independent donors. Specific synaptic and nonsynaptic masks were applied to quantify the synaptic enrichment of F-actin and HLA-A, -B, and -C molecules in target cells, as described previously (42) (*SI Appendix*, Fig. S3). Cancer cells with a polarized actin cytoskeleton showed a significantly higher frequency and magnitude of HLA-A, -B, and -C enrichment at the IS compared to those lacking F-actin polarization (*P* < 0.001, n = 100 target cells per phenotype; Fig. 2 *B* and *C*). This relationship was further supported by strong correlation values between synaptic F-actin polarization and HLA-A, -B, and -C accumulation (R = 0.7 and R = 0.8 for NK cells from donor #4 and #5, respectively, *P* < 0.001; Fig. 2*D*). Interestingly, the overall surface levels of HLA-A, -B, and -C molecules remained comparable between target cells with and without synaptic actin polarization (Fig. 2*E*), suggesting that synaptic enrichment results from the redistribution of preexisting surface molecules. To assess whether this redistribution depends on interactions with cognate NK cell receptors, we repeated the analysis using iKIR-deficient NK-92MI cells as effector cells. Both confocal microscopy and IFC yielded similar results to those obtained with primary NK cells (*SI Appendix*, Fig. S4). Specifically, HLA-A, -B, and -C molecules accumulated prominently at the IS in target cells exhibiting synaptic F-actin polarization, whereas their distribution remained uniform in target cells lacking this phenotype (*SI Appendix*, Fig. S4 *A*–*D*). Notably, a strong correlation was also observed between synaptic F-actin polarization and HLA-A, -B, and -C accumulation in assays with NK-92MI, reaching 0.86 (*P* < 0.001; Fig. 4*E*). To assess whether synaptic redistribution also applies to another type of regulatory ligands, we examined the localization of MICA and MICB, two ligands for the activating receptor NKG2D. Using confocal microscopy, we selected conjugates formed with either primary NK cells or NK-92MI cells that exhibited strong synaptic F-actin polarization within the cancer cell, along with detectable surface levels of MICA/B. We then compared, within the same cancer cells, the synaptic distribution of MICA/B to that of HLA-A, -B, and -C (*SI Appendix*, Fig. S5*A*). Despite robust actin polarization in the target cell, MICA/B did not exhibit strong or consistent enrichment at the IS, in contrast to the pronounced accumulation observed for HLA-A, -B, and -C molecules (*SI Appendix*, Fig. S5*B*). This was confirmed by nonsignificant correlation values between synaptic F-actin polarization and MICA/B accumulation (*SI Appendix*, Fig. S5*C*). These findings, consistent across both NK cell types, suggest that actin-associated redistribution of surface molecules toward the IS in cancer cells does not uniformly apply to all ligand types.

To determine whether our findings on HLA-A, -B, and -C extend beyond MDA-MB-231 cells, we examined the synaptic abundance of these molecules in MDA-MB-468 cells, a more epithelial-like and EGFR-dependent breast carcinoma cell line. When MDA-MB-468 cells were engaged by either primary NK cells or NK-92MI cells, synaptic F-actin polarization similarly promoted HLA-A, -B, and -C accumulation at the IS, confirming that this phenomenon is not limited to MDA-MB-231 cells (*SI Appendix*, Fig. S6 *A*, *B*, *D*, and *E*). Moreover, in these cells as well, overall HLA-A, -B, and -C surface expression remained unchanged regardless of synaptic actin polarization (*SI Appendix*, Fig. S6 *C* and *F*), further supporting the idea that IS enrichment results from redistribution rather than increased expression.

Together, these findings indicate a strong association between synaptic polarization of the cancer cell actin cytoskeleton and the localized enrichment of HLA-A, -B, and -C molecules at the IS. This enrichment appears to occur independently of inhibitory KIR interactions, as demonstrated by its persistence in assays with KIR-deficient NK-92MI cells, and is driven by the polarization of preexisting surface molecules toward the IS.

### Polarization of HLA Inhibitory Ligands to the IS Requires a Functional Actin Cytoskeleton.

**Fig. 3.**
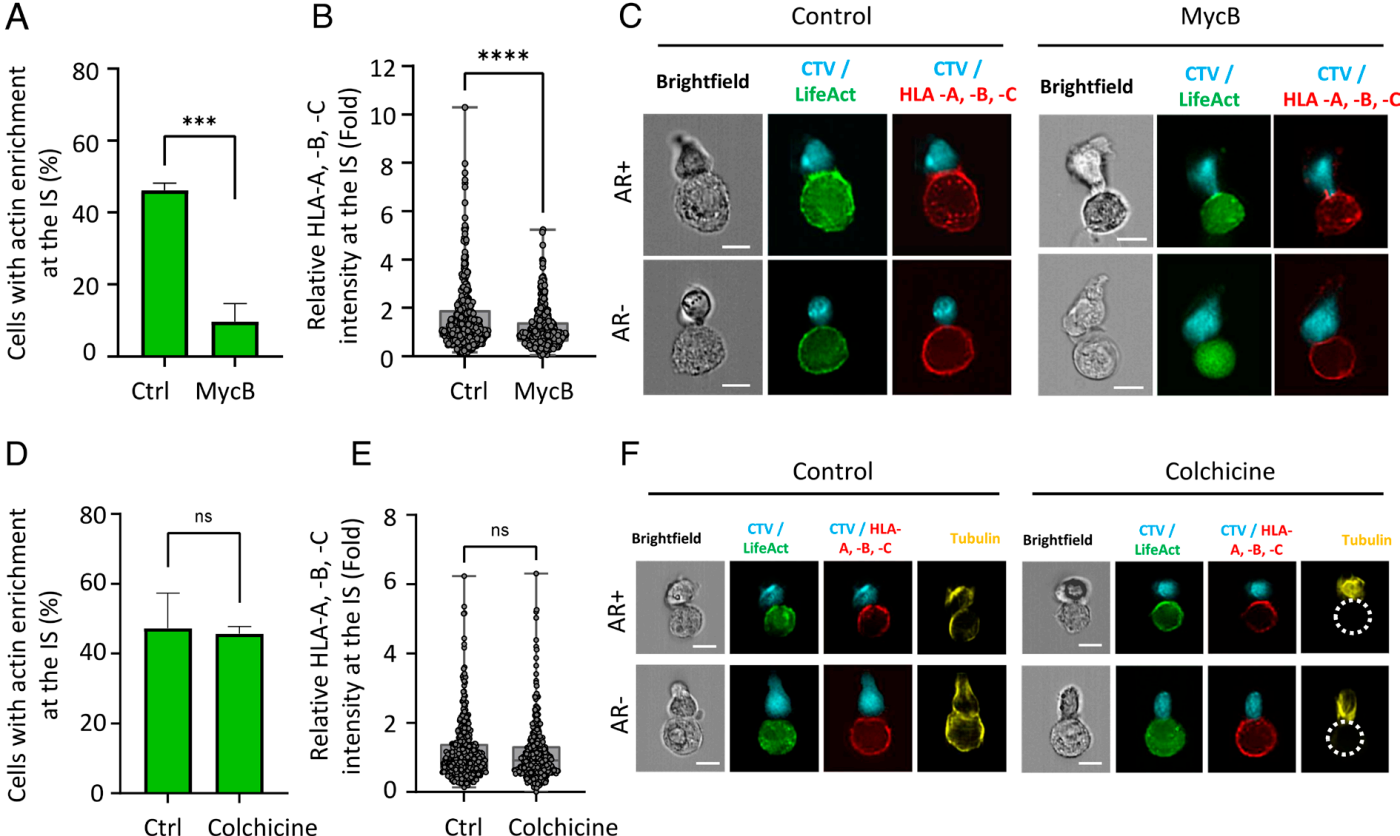
Synaptic polarization of HLA-A, -B, and -C depends on the actin cytoskeleton, not microtubules. Emerald-LifeAct-expressing MDA-MB-231 cells (green) were treated with cytoskeletal-disrupting agents and labeled for HLA-A, -B, and -C (red), conjugated with CTV-stained NK-92MI cells (cyan), and analyzed by IFC. Data were collected from three independent experiments, with n = 200 cell-to-cell conjugates analyzed per condition. (*A*–*C*) Cancer cells were treated with mycalolide B (MycB, an inhibitor of actin filament polymerization) at a concentration of 1 µM for 20 min. (*A*) Relative Emerald-LifeAct intensity at the IS was quantified, and the percentage of cell-to-cell conjugates with synaptic actin cytoskeleton remodeling (ratio >1) was calculated. (*B*) Relative HLA-A, -B, and -C intensities at the IS in cell-to-cell conjugates. (*C*) Representative IFC images of cell-to-cell conjugates, with or without synaptic actin cytoskeleton remodeling (AR+ and AR−, respectively). (Scale bars, 10 µm.) (*D*–*F*) Cancer cells were treated with colchicine (a microtubule-disrupting agent) at a concentration of 2.5 µM for 90 min. (*D*) Relative Emerald-LifeAct intensity at the IS was quantified, and the percentage of cell-to-cell conjugates with synaptic actin cytoskeleton remodeling was calculated. (*E*) Relative HLA-A, -B, and -C intensities at the IS in cell-to-cell conjugates. (*F*) Representative IFC images of cell-to-cell conjugates, with or without synaptic actin cytoskeleton remodeling (AR+ and AR−, respectively). Dashed lines outline the target cell as microtubules are not visible following colchicine treatment. (Scale bars, 10 µm.) Statistical significance was determined using the Mann–Whitney test.

The role of the actin cytoskeleton in the polarization of HLA-A, -B, and -C molecules to the IS was investigated using mycalolide B (MycB), a marine macrolide toxin that depolymerizes F-actin into monomeric actin and irreversibly disrupts actin dynamics (43). Treatment with 1 µM MycB resulted in extensive F-actin depolymerization in MDA-MB-231 cells (*SI Appendix*, Fig. S7*A*). Following MycB pretreatment and subsequent drug removal, MDA-MB-231 cells were cocultured with NK cells as previously. Quantitative analysis using IFC revealed a marked reduction in the percentage of MycB-treated cells exhibiting actin cytoskeleton polarization during NK cell interactions, decreasing from approximately 45% in DMSO-treated control cells to less than 10% (*P* < 0.001; n = 3) (Fig. 3*A*). This disruption of synaptic actin remodeling led to a statistically significant and substantial decrease in the synaptic enrichment of HLA-A, -B, and -C molecules compared to control cells (*P* < 0.0001; Fig. 3 *B* and *C*). Of note, MycB treatment reduced the conjugation rate by approximately 50% (*SI Appendix*, Fig. S7*C*), highlighting the requirement for an intact target cell cytoskeleton in supporting NK cell adhesion. To verify that these effects were specific to actin cytoskeleton disruption, additional experiments were conducted using colchicine, a microtubule-disrupting agent. Treatment of MDA-MB-231 with 2.5 µM colchicine effectively disrupted microtubules (*SI Appendix*, Fig. S7*B*) but had no measurable impact on the proportion of cells displaying synaptic actin polarization during NK cell interactions, which remained around 45% (Fig. 3*D*). Furthermore, unlike MycB, colchicine did not significantly impair the enrichment of HLA-A, -B, and -C molecules at the IS (Fig. 3 *E* and *F*), nor did it affect the conjugation rate (*SI Appendix*, Fig. S7*C*). These findings underscore the critical and specific role of the actin cytoskeleton in mediating the recruitment of HLA-A, -B, and -C molecules to the IS.

To evaluate whether HLA class I expression, in turn, influences actin remodeling in target cells, we used CRISPR-Cas9 to knock out β2-microglobulin (B2M), a key component required for surface expression of HLA class I molecules. Flow cytometry confirmed the complete loss of HLA class I at the cell surface in B2M-deficient MDA-MB-231 cells (*SI Appendix*, Fig. S8*A*). These cells were then cocultured with either NK92MI or primary NK cells, and their capacity to undergo synaptic actin remodeling was assessed. Quantitative analysis revealed no significant difference in the frequency of actin remodeling between B2M-deficient and control cells (*SI Appendix*, Fig. S8 *B*–*E*), indicating that cytoskeletal polarization occurs independently of HLA class I expression.

### Actin Cytoskeleton Remodeling–Driven Synaptic Polarization of Inhibitory Ligands Enhances Inhibitory Signaling Toward NK Cells.

Based on our findings, we hypothesized that inhibition of NK cell polarization in front of target cells exhibiting synaptic F-actin polarization results from the recruitment and local accumulation of HLA inhibitory ligands, which in turn enhances inhibitory signaling toward NK cells. To test this hypothesis, we investigated the interplay between synaptic actin cytoskeleton remodeling and HLA inhibitory ligand polarization in mediating NK cell suppression, employing an HLA-blocking antibody to disrupt iKIR signaling. The HLA-blocking antibody was first validated to ensure that the localization pattern of HLA molecules, including their synaptic accumulation in cancer cells with F-actin synaptic polarization, remained consistent with the observations made using nonblocking antibodies (*SI Appendix*, Fig. S9).

**Fig. 4.**
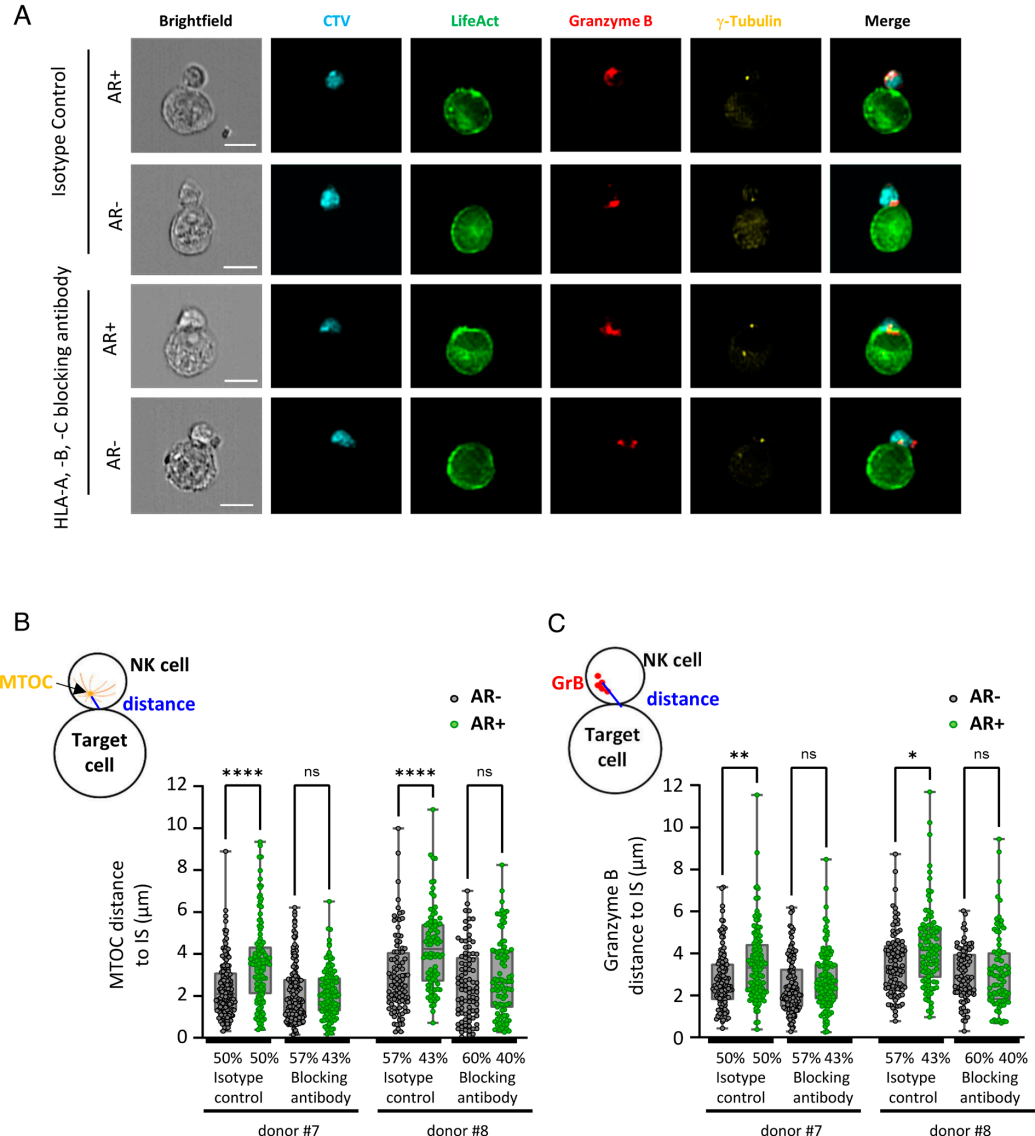
HLA-blocking antibody counteracts synaptic actin remodeling–driven inhibition of NK cell polarization. Emerald-LifeAct-expressing MDA-MB-231 cells (green) were pretreated with either an HLA-A, -B, and -C blocking antibody or an isotype control antibody before being conjugated with CTV-labeled primary NK cells (cyan) for 60 min. After conjugation, cells were immunolabeled for Granzyme B and γ-Tubulin and analyzed using IFC. Data were collected from NK cells isolated from two distinct donors, with n = 100 cell-to-cell conjugates analyzed per condition. (*A*) Representative IFC images of cell-to-cell conjugates between primary NK cells and MDA-MB-231 cells, with or without synaptic actin cytoskeleton remodeling (AR+ and AR−, respectively). (Scale bar, 10 µm.) (*B* and *C*) Emerald-LifeAct relative intensity at the IS was used to classify MDA-MB-231 cells into AR+ and AR− groups (ratio >1 and ratio <1, respectively). The percentage of conjugates of each subgroup is indicated for each condition. NK cell lytic machinery polarization was evaluated by measuring the distance between the MTOC and the synapse center (*B*) and the distance between the Granzyme B centroid and the synapse center (*C*). Distances for AR+ (green spots) and AR− (gray spots) subgroups are presented. Statistical significance was determined using the Kruskal–Wallis test.

MDA-MB-231 cells were preincubated with the HLA-blocking antibody or an IgG isotype control before coculture with primary NK cells. Polarization of the MTOC and cytotoxic granules in NK cells derived from two different donors was quantified using IFC (Fig. 4). HLA blockade, but not the IgG control, significantly increased the polarization of both the MTOC and cytotoxic granules in NK cells interacting with target cells exhibiting synaptic actin cytoskeleton remodeling. Notably, under these conditions, NK cell polarization was restored to levels comparable to those observed in interactions with target cells lacking actin cytoskeleton remodeling (Fig. 4 *B* and *C*). We extended our analysis to MDA-MB-468 cancer cells, where HLA blockade similarly reversed actin cytoskeleton–driven inhibition of NK cell polarization (*SI Appendix*, Fig. S10). These findings reinforce and expand upon previous observations, supporting that the protective effect of F-actin synaptic polarization in cancer cells against NK cell cytotoxicity is mediated by inhibitory signaling through iKIR engagement with HLA molecules.

To further determine whether actin cytoskeleton remodeling in cancer cells impairs NK cell activation through inhibitory ligand–receptor interactions, we employed matched and mismatched iKIR-ligand models. NK cells from donors were isolated and sorted into subpopulations either expressing or lacking KIR2DL1 (*SI Appendix*, Fig. S11*A*), an iKIR that recognizes group 2 HLA-C (HLA-C2) molecules expressed by MDA-MB-231 cells (44). Both subpopulations (KIR2DL1+ and KIR2DL1−) were expanded to ensure sufficient cell numbers for subsequent analyses. The expression of HLA-C2 on the surface of MDA-MB-231 cells was confirmed using a specific antibody (*SI Appendix*, Fig. S11*B*). Notably, HLA-C2 expression was found to be relatively low compared to the overall expression of HLA-A, -B, -C (*SI Appendix*, Fig. S11 *B* and *C*). HLA-C was confirmed to polarize to the synaptic region in NK cell–conjugated target cells exhibiting synaptic F-actin accumulation, whereas it remained uniformly distributed on the surface of target cells lacking actin cytoskeleton remodeling (*SI Appendix*, Fig. S11*D*).

**Fig. 5.**
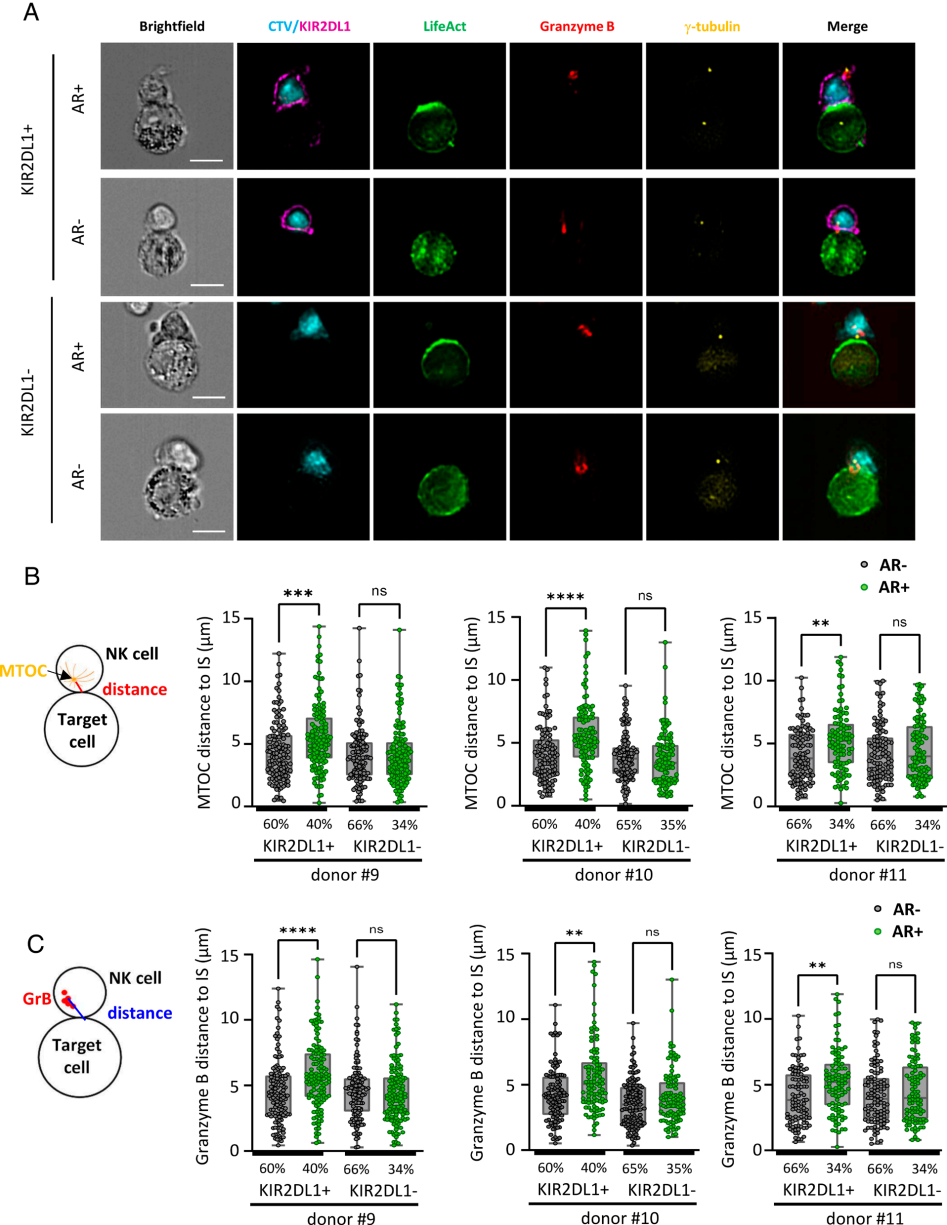
Inhibition of NK cell polarization by cognate HLA–iKIR interactions is associated with synaptic actin polarization. Emerald-LifeAct-expressing MDA-MB-231 cells (green) were cocultured for 60 min with CTV-labeled primary NK cells, either KIR2DL1^+^ or KIR2DL1^−^ cells. After coculture, cells were immunolabeled for Granzyme B (red), γ-Tubulin (yellow), and KIR2DL1 (magenta). Data were collected from NK cells isolated from three distinct donors, with n = 100 cell-to-cell conjugates analyzed per condition. (*A*) Representative IFC images of cell-to-cell conjugates between primary NK cells (KIR2DL1^+^ or KIR2DL1^−^) and MDA-MB-231 cells, with or without synaptic actin cytoskeleton remodeling (AR+ and AR−, respectively). (Scale bars, 10 µm.) (*B* and *C*) Emerald-LifeAct relative intensity at the synapse was used to classify MDA-MB-231 cells into AR+ and AR− groups (ratio >1 and ratio <1, respectively). The percentage of conjugates of each subgroup is indicated for each condition. NK cell lytic machinery polarization was evaluated by measuring the distance between the MTOC and the synapse center (*B*) and the distance between the Granzyme B centroid and the IS center (*C*). Distances for AR+ and AR− subgroups are presented. Statistical significance was determined using the Kruskal–Wallis test.

IFC was used to quantitatively assess MTOC and cytotoxic granule polarization in KIR2DL1− and KIR2DL1+ NK cells from three different donors. NK cells from each subpopulation were analyzed during interactions with target cells either exhibiting or lacking actin cytoskeleton remodeling (Fig. 5). KIR2DL1− NK cells demonstrated comparable MTOC and cytotoxic granule polarization regardless of the target cell’s cytoskeletal phenotype. In contrast, KIR2DL1+ NK cells exhibited polarization similar to that of KIR2DL1− NK cells when interacting with target cells lacking actin cytoskeleton remodeling but showed significantly reduced polarization when engaging target cells with synaptic F-actin accumulation. These findings indicate that actin-driven polarization of inhibitory ligands to the cancer cell side of the IS strengthens inhibitory signaling toward interacting NK cells, leading to suppression of NK cell polarization.

## Discussion

MHC-I downregulation is a well-established mechanism by which tumor cells evade cytotoxic T cell responses and resist immunotherapy (45). Paradoxically, this reduction in MHC-I expression increases their susceptibility to NK cell attack, as MHC-I molecules play a crucial role in suppressing NK cell activation (7). Our findings reveal that cancer cells can counteract this vulnerability by redistributing their limited MHC-I pool to the IS through actin remodeling. By locally concentrating MHC-I at the IS, cancer cells amplify inhibitory signaling to interacting NK cells, effectively mimicking higher MHC-I expression and thereby dampening NK cell activation. Notably, a similar mechanism has been described in mature dendritic cells (DCs), which engage NK cells to stimulate them (46) while avoiding destruction (47). In this so-called regulatory synapse (48), F-actin polymerization at the DC interface facilitates MHC-I accumulation, reinforcing inhibitory signaling and suppressing NK cell cytotoxicity. Our findings suggest that cancer cells exploit this protective mechanism, originally evolved to modulate immune responses, as a mean to evade NK cell–mediated killing. We demonstrate that even a relatively low abundance of MHC-I molecules, such as HLA-C2 on MDA-MB-231 cells, can amplify inhibitory signaling in a measurable manner when strategically concentrated at the synapse—precisely at the optimal location and timing during NK cell interactions.

Recent studies have identified the target cell’s actin cytoskeleton as a key regulator of cytotoxic lymphocyte activity by modulating target cell rigidity and deformability (34, 38). These biophysical properties contribute to the mechanical forces at the IS, which influence force-sensitive receptors on cytotoxic lymphocytes, fine-tuning immune responses through a process known as mechanosurveillance (38). Our findings extend this understanding by demonstrating that, beyond shaping the biophysical properties of target cells, the actin cytoskeleton directly regulates the molecular composition of the IS by controlling the synaptic density and distribution of key surface molecules. Interestingly, the synaptic polarization of HLA molecules is strictly actin-dependent and independent of microtubules. In addition, this process occurs irrespective of the engagement of HLA molecules with their cognate receptors on NK cells, as evidenced by the persistence of F-actin polarization even when HLA–iKIR interactions are pharmacologically inhibited or when NK92-MI cells, which lack iKIRs, are used. Therefore, actin dynamics emerge as the primary driver in redirecting inhibitory signaling toward the IS. While directly inhibiting actin dynamics is not a viable therapeutic approach due to its high toxicity and widespread cellular effects, targeting the regulatory pathways controlling rapid F-actin remodeling in target cells could offer opportunities for enhancing NK cell–mediated anti-tumor responses. Additionally, neutralizing these pathways through CAR-NK cell engineering could improve therapy efficacy by offering a more specific alternative to broadly overriding inhibitory signaling, which carries a higher risk of off-target toxicity (49). Identifying the molecular trigger responsible for actin remodeling is therefore a critical next step toward the translation of our findings into therapeutic strategies.

At the same time, dissecting the functional consequences of this remodeling is essential to fully understand how it shapes immune interactions. A key question in this regard is how synaptic actin remodeling mediates local increase in MHC-I surface density. At the DC-NK cell regulatory synapse, F-actin has been proposed to stabilize the low-affinity interaction between MHC-I and KIRs (47). By analogy to its role in regulating MHC-II diffusion (50), the actin-based membrane skeleton may create membrane compartments or “fences” that constrain molecular mobility, thereby increasing MHC-I concentration at the synapse (47). Since our data indicate that the overall MHC-I surface expression does not significantly differ between target cells with or without polarization, we also favor a model in which the actin cytoskeleton regulates the distribution of preexisting MHCI molecules, e.g., by restricting their diffusion away from the synaptic region, rather than facilitating their targeted delivery to the synapse.

Beyond clustering inhibitory ligands, emerging evidence suggests that actin remodeling at the cancer cell side of the IS plays a broader role in reconfiguring intracellular trafficking. Specifically, actin dynamics have been recently implicated in the enrichment and fusion of exosome-containing multivesicular bodies at the IS (51), suggesting that this interface functions as a polarized secretory domain. While the role of targeted secretion from the cancer cell side of the IS remains to be fully elucidated, it may contribute to immune evasion by directing exosomes enriched in MHC-I and immune checkpoint molecules toward NK cells, thereby reinforcing inhibitory signaling and sustaining NK cell suppression. Other recent studies have further emphasized the dynamic nature of the cancer cell side of the IS. In melanoma cells, a calcium-dependent membrane repair response is rapidly activated following sublethal hits by CD8+ T cells, involving lysosome mobilization to the IS to counteract cytotoxicity (52). In breast cancer cells under NK cell attack, the synaptic membrane composition undergoes remodeling, characterized by an increase in densely packed lipids, which reduces perforin binding and enhances resistance to NK cell killing (53). Given the well-established roles of actin in regulating membrane trafficking, it is worth investigating whether synaptic actin remodeling directly orchestrates these defense mechanisms, potentially serving as a point of convergence that could be targeted to simultaneously inhibit multiple evasion strategies.

Collectively, our findings reveal a deeper level of organization at the IS, suggesting that in its resistant state, the cancer cell side of the IS mirrors the lymphocyte’s side, with each cell actively polarizing its molecular arsenal for either attack or defense. Just as cytotoxic lymphocytes undergo actin-driven polarization to coordinate effector functions, cancer cells seem to exploit cytoskeletal remodeling to reinforce inhibitory signaling, repair and modify the membrane, and potentially direct immunosuppressive secretions. These insights emphasize the IS as a dynamic battlefield, where the structural and molecular adaptations of each interacting cell ultimately dictate the outcome.

## Methods

### Cell Lines and Cell Culture.

MDA-MB-231 (ATCC HTB-26), MDA-MB-468 (ATCC HTB-132), and NK-92MI (ATCC CRL-2408) cell lines were purchased from the American Type Culture Collection (ATCC). Breast cancer cell lines were transduced to stably express the mEmerald-Lifeact-7 F-actin reporter (Addgene, plasmid #54148), as previously described (39). MDA-MB-231 and MDA-MB-468 cells were maintained in Dulbecco’s Modified Eagle Medium (DMEM) supplemented with 10% fetal bovine serum (FBS), 100 U/mL penicillin, and 100 μg/mL streptomycin. NK-92MI cells were grown in RPMI-1640 medium supplemented with 10% FBS, 10% horse serum, 100 U/mL penicillin, and 100 μg/mL streptomycin. All cell lines were authenticated and verified to be free of cross-contamination via short tandem repeat (STR) profiling (Microsynth). Cells were cultured in a humidified incubator at 37 °C with 5% CO_2_’ and were routinely screened for *Mycoplasma* contamination.

B2M knockout cell lines were generated by electroporating MDA-MB-231 cells stably expressing Emerald-LifeAct. Prior to electroporation, 1 × 10^5^ cells were harvested and resuspended in electroporation buffer. A CRISPR Gene Knockout Kit targeting the human B2M gene (EditCo Bio) was employed. Ribonucleoprotein (RNP) complexes were assembled by incubating 120 pmol of *Streptococcus pyogenes* Cas9 (spCas9) protein with a pool of three single-guide RNAs (sgRNAs; 150 pmol each), each targeting a distinct locus within the B2M gene. The sgRNA sequences used were CGGAGCGAGAGAGCACAGCG, GGCCGAGATGTCTCGCTCCG, and ACTCACGCTGGATAGCCTCC. The resuspended cells were combined with the preassembled Cas9/sgRNA RNP complex and electroporated using the Neon Electroporation System (Thermo Fisher Scientific) under the following parameters: 1,300 V, 20 ms pulse width, 3 pulses. Immediately following electroporation, cells were transferred into complete growth medium and cultured to allow for recovery. Knockout efficiency was evaluated by PCR and flow cytometry.

### Donor Population Characteristics, Isolation of Human Primary NK Cells, and Amplification of the KIR2DL1^+/−^ Subpopulations.

Buffy coats were provided by the Luxembourg Red Cross Blood Transfusion Centre (CTS, Luxembourg-City) from healthy, unpaid, and volunteer donors. According to the CTS 2024 activity report, 15,588 individuals were registered as active donors (46.2% women, 53.8% men), and 93.1% of them were listed as whole-blood donors. During the same year, the 17,624 allogeneic whole-blood donations were collected. The mean age of active donors was 42.7 y, with a broad distribution: Roughly one-fifth were 18 to 30 y old, one-quarter 31 to 40 y, one-quarter 41 to 50 y, and one-quarter 51 to 60 y, with the remainder aged 61 to 70 y. Written informed consent was obtained by the CTS before each donation, and residual buffy-coat fractions were anonymized at source. No individual demographic, ethnic, or genetic identifiers accompanied the material provided to researchers ensuring compliance with the general data protection regulation (GDPR).

Primary NK cells were isolated from cryopreserved peripheral blood mononuclear cells (PBMCs) obtained from buffy coats. NK cell isolation was performed using the human NK cell isolation kit (Miltenyi Biotec #130-092-657) via negative selection, according to the manufacturer’s instructions. Briefly, PBMCs were incubated with biotin-conjugated monoclonal antibodies against antigens not expressed by NK cells, followed by a second incubation with microbeads conjugated to monoclonal antibodies. Labeled cells were subsequently separated using a magnetic column system. After isolation, the NK cells were washed and resuspended in prewarmed RPMI medium supplemented with 10% FBS, 1% HEPES (10 mM), 1% MEM-NEAA, 1% sodium pyruvate, 100 U/mL penicillin, and 100 μg/mL streptomycin, IL-2 (100 IU/mL, Miltenyi Biotec #130-097-746) and IL-15 (10 ng/mL, Miltenyi Biotec #130-095-765). The cells were adjusted to a final concentration of 2 × 10^6^ cells/mL and incubated overnight before being used in experiments.

For live imaging experiments, isolated NK cells were washed and resuspended in 60% DMEM, 25% F-12, 10% human serum, 1% HEPES (10 mM), 1% MEM-NEAA, 1,100 U/mL penicillin, 100 μg/mL streptomycin, and IL-2 (500 IU/mL, Miltenyi Biotec #130-097-746). NK cells were seeded in 24-well plates at a density of 1 × 10^6^ cells/well. Cells were maintained for up to 2 wk, with splitting and addition of fresh medium containing cytokines every 3 d, prior to use in downstream experiments.

In a subset of experiments, isolated human primary NK cells were stained to identify, isolate, and amplify KIR2DL1-expressing cells. Live NK cells were identified by incubating them with the Zombie NIR fixable viability kit (BioLegend #423106) for 15 min at room temperature. After washing in MACS buffer (Miltenyi Biotec #130-091-221), Fc receptor blocking solution (Human TruStain FcX, BioLegend #422302) was added for 10 min to minimize nonspecific binding. Cells were then washed and incubated for 30 min in the following antibody staining mix: FITC-conjugated anti-CD56 antibody (BioLegend #318304), PE-conjugated anti-CD3 antibody (BioLegend #300330), and PE-conjugated anti-CD158a (KIR2DL1) antibody (Miltenyi #130-120-446). KIR2DL1^+^ and KIR2DL1^−^ NK cells were sorted using a fluorescence-activated cell sorter (BD FACSymphony^TM^ S6 lite). Sorted NK cells were seeded in 96-well U-bottom plates at a density of 8 × 10^4^ cells/well in 200 µL of complete medium containing 60% DMEM, 25% F-12, 10% human serum, 1% HEPES (10 mM), 1% MEM-NEAA, 100 U/mL penicillin, 100 μg/mL streptomycin, IL-2 (400 IU/mL, Miltenyi Biotec #130-097-746), and PHA-P (1 µg/mL, Invivogen #inh-phap). To facilitate cell expansion, the NK cells were cocultured with 1 × 10^5^ of PBMCs per well from two distinct donors. To block PBMCs proliferation, they were pretreated with mitomycin C (20 µg/mL, Merck #M4287-2MG) during 30 min at 37 °C in complete media prior to coculture with NK cells. Cells were split every 3 d over a 2-wk period before use in downstream experiments.

### Live Cell Imaging.

Primary NK cells were stained with CMRA (Invitrogen #C34551, 1:1,000 dilution) in serum-free medium for 20 min at 37 °C. Emerald-LifeAct-expressing MDA-MB-231 cells were detached and seeded at 8 × 10^4^ cells per well on poly-D-lysine-coated 8-well slides (ibidi) 5 min before imaging. Cells were maintained in Leibovitz’s L-15 medium (no phenol red, Gibco #21083027) containing 1 µM SYTOX Blue dead cell stain (Invitrogen #S34857). Slides were mounted on a heated stage within a temperature-controlled chamber maintained at 37 °C with 5% CO_2_. At the start of the recording, 8 × 10^4^ pNK cells were added. Image acquisition was performed on a Zeiss LSM880 confocal microscope using ZEN Black software.

### Fluorescence Staining and Preparation of Cell-to-Cell Conjugates for Confocal Microscopy.

NK cells were stained with CellTracker Deep Red dyes (Invitrogen #C34565, 1:1,000 dilution) in serum-free medium for 20 min at 37 °C. Ligand labelling was performed in MACS buffer (Miltenyi Biotec #130-091-221) using PE-conjugated anti-MICA/MICB (Mouse IgG2a, BioLegend #320906), APC-conjugated anti-HLA-A, -B, and -C (Mouse IgG1, BD Pharmingen # 562006), PE-conjugated anti-HLA-A, -B, and -C (BD Pharmingen #560964) or PE-conjugated anti-HLA-C (BD Pharmingen #566372) antibodies for 30 min at 4 °C. For conjugate formation, equal numbers of labeled NK cells (7 × 10^4^ in 100 µL) and cancer cells (7 × 10^4^ in 100 µL) were mixed and incubated at 37 °C for 40 min (for HLA localization) or 60 min (for NK activation) in the presence of Hoechst 33342 (1:2,000, Miltenyi Biotec #130-111-569). Conjugates were then transferred onto poly-L-lysine-coated µ-slide chambers (Ibidi #80806) and allowed to adhere for 8 min. Cells were fixed with 2% paraformaldehyde (PFA, Agar Scientific) for 15 min at room temperature and permeabilized with 0.1% Triton X-100 for 5 min for intracellular labelling. Following a 1-h blocking step in PBS containing 5% BSA, 2% FBS, and 5% NGS, cells were incubated with primary antibodies against Granzyme B (BioLegend #396406) and γ-Tubulin (Santa Cruz Biotechnology #sc-17788) for 2 h at room temperature. After washing, secondary antibodies (Alexa Fluor-conjugated goat anti-mouse, anti-mouse IgG2a, anti-rabbit, or anti-rat, e.g., AF555 or AF647, Invitrogen) were applied. Finally, cells were washed, and the medium was replaced with mounting medium (Ibidi #50001) for imaging. In some experiments, adherent target cells were pretreated with mycalolide B (1 µM, Enzo #BML-T123-0020) for 20 min, colchicine (2.5 µM, Merck #C9754) for 1.5 h, or DMSO as a control. After fixation and permeabilization, cells were stained with an AF647-conjugated anti-α-Tubulin antibody (BioLegend #627908) to assess drug effects.

### Confocal Imaging and Quantitative Image Analysis.

Images were acquired using a Zeiss LSM880 fast Airyscan confocal microscope with excitation lasers (405, 488, 543, 594, and 633 nm) in a multitrack setup. Acquisitions were performed in confocal or Airyscan mode, with z-stacks (0.5 or 0.2 μm interval). Image type (confocal/Airyscan) is specified in the figure legends. For Granzyme B analysis, a z-stack of the entire NK cell was acquired (0.5 μm intervals, ~20 slices). Quantification was performed using ImageJ v1.53t. Granzyme B polarization was assessed using a Z-projection (“sum slices” method) as previously described (51). A region of interest (ROI) encompassing the target cell was manually segmented, with the synaptic region defined as the one-third portion in direct contact with the NK cell. Granzyme B localization was quantified as the ratio of mean pixel intensity (MPI) in the synaptic ROI to the total ROI. To measure MTOC positioning relative to the synapse, coordinates of the MTOC and synapse center were obtained using the “multipoint tool,” and distances were calculated as d = $\sqrt{({x2-x1)}^{2}+({y2-y1)}^{2}}$.

For Emerald-LifeAct, HLA-A, -B, and -C and MICA/MICB fluorescence profiling, a 100-pixel (3.5 µm) line for NK-92MI and a 50-pixel line for primary NK (pNK) cells was drawn across conjugates, and fluorescence intensity was analyzed using the “Plot Profile” tool. Enrichment was determined as the ratio of maximum intensity at the IS to the maximum intensity at the opposite side of the target cell. Conjugates were classified as actin remodeling-positive (AR+) or -negative (AR−) based on the relative Emerald-LifeAct intensity at the IS, with AR+ defined by a ratio >1 and AR- by a ratio <1.

### Fluorescence Staining and Preparation of Cell-to-Cell Conjugates for IFC.

NK cells were stained with CellTrace Violet (CTV, Thermo Fisher Scientific #C34557, 1:8,000) and Zombie NIR (BioLegend #423105, 1:2,000) for 20 min in PBS before conjugation. Alternatively, NK cells were stained with PE/Cy7-conjugated anti-CD56 (BioLegend #318318) and Hoechst for 30 min at 4 °C in MACS buffer (Miltenyi Biotec #130-092-987) before conjugation. For extracellular labelling, cells were incubated in MACS buffer with PE/Dazzle594-conjugated anti-HLA-A, -B, and -C (BioLegend #311410), or PE-conjugated anti-CD158a (BioLegend #374904) for 30 min at 4 °C. Target and effector cells were conjugated at a 1:1 ratio (5 × 10^5^ NK cells and 5 × 10^5^ MDA-MB-231 cells in 200 µL). After 40 min (for HLA localization) or 60 min (for NK activation), cells were fixed with 4% PFA, permeabilized with 0.1% Triton X-100, and stained with AF594-conjugated anti-γ-Tubulin (Santa Cruz Biotechnology #sc-17788) and APC-conjugated anti-Granzyme B (BioLegend #396408). Cells were resuspended in 35 µL PBS for acquisition. For HLA-A, -B, and -C blockade, MDA-MB-231 cells were preincubated with Ultra-LEAF™ anti-HLA-A, -B, and -C (BioLegend #311428) or an isotype control (BioLegend #400264) at 50 µg/mL for 30 min, followed by coculture with primary NK cells. Fixation and intracellular staining for Granzyme B and MTOC followed. To verify antibody fixation, one sample was stained with goat anti-mouse AF555. For cytoskeleton studies, target cells were detached and treated with mycalolide B (Enzo #BML-T123-0020, 1 µM, 20 min), colchicine (Merck #C9754, 2.5 µM, 1.5 h), or DMSO. Posttreatment, cells were washed and stained for HLA-A, -B, and -C. Conjugation time was reduced to 30 min for colchicine due to the reversible nature of colchicine’s effects.

Assessment of apoptosis markers has been described before (39, 40). In short, primary NK cells were stained with CellTrace Violet (CTV, Thermo Fisher Scientific #C34557, 1:8,000) and Zombie NIR (BioLegend #423105, 1:2,000) for 20 min in PBS before conjugation. Cell conjugates were allowed to form for 50 min before centrifugation and staining with Apotraker-AF647 (dilution 1:250, BioLegend, #427405) and Zombie Red (1:1,500, BioLegend, #423109) in staining buffer (BioLegend, #420201) for 15 min at RT. After very gentle washing, conjugate were fixed with 2% PFA, washed, and resuspended in 35 µL PBS for acquisition.

### IFC and Quantitative Image Analysis.

Samples were acquired using an ImageStream®X Mark II (Cytek Biosciences) with four lasers (405, 488, 561, and 642 nm) and operated using INSPIRE® software. Single-color controls were used for matrix compensation. A total of 5 × 10^4^ to 8 × 10^4^ conjugates were acquired per sample at 60X magnification. IFC analysis was conducted using IDEAS software, following Biolato et al. (42). Briefly, the synaptic mask was generated at the NK–target interface and extended into the target cell (~1/3 of total target area). A nonsynaptic mask covering the remaining portion of the cell was also created. MPI was calculated within each mask, and F-actin enrichment was determined as the ratio of MPI in the synaptic mask to the nonsynaptic mask. Cells were categorized as AR− (ratio <1) or AR+ (ratio >1), with classification validated through visual inspection. The same analysis was applied to ligand enrichment, restricting masks to the cell membrane. MTOC and Granzyme B polarization were assessed using the “delta centroid” feature, calculating the distance between the centroid of the MTOC or Granzyme B mask and the synaptic mask centroid.

### HLA Expression Analysis by Flow Cytometry.

MDA-MB-231 cells were labeled with the Zombie NIR viability kit (BioLegend #423106, 15 min, RT). After washing, cells were incubated with PE-conjugated anti-HLA-A (BD Pharmingen #567739), PE-conjugated anti-HLA-B (BD Pharmingen #567211), PE-conjugated anti-HLA-C (BD Pharmingen #566372), PE-conjugated anti-HLA-A,-B,-C (BD Pharmingen #560964), or their respective isotype controls: PE-Mouse IgG1 (BD Pharmingen #554680), PE-Mouse IgG2b (BD Pharmingen #555058), PE-Rat IgG1 (BioLegend #400408) for 30 min at 4 °C in the dark. Samples were analyzed using an ID7000™ Spectral flow cytometer (Sony), and HLA expression was assessed using FlowJo 10.8.1 (BD) software.

### Analysis of the Conjugation Rate by Flow Cytometry.

Conjugation rate between NK and tumor cells was assessed using flow cytometry. NK-92MI and primary NK cells were stained with CellTrace Violet (CTV) (1:6,000, Thermo Fisher Scientific, #C34557) and Zombie NIR (1:400), while Emerald-LifeAct expressing target cells were stained with Zombie NIR (1:400) in PBS for 20 min at RT. Target and effector cells were conjugated at a 2:1 ratio (1 × 10^5^ NK cells and 2 × 10^5^ MDA-MB-231 cells) in 200 µL and incubated for 40 min at 37 °C. Following incubation, 300 µL of ice-cold 0.5% paraformaldehyde (PFA) in PBS was added to fix the cells prior to analysis on an ID7000™ Spectral Cell Analyzer (Sony). Unstained and single-stained controls were collected for each experiment. The data generated were analyzed with the FlowJo 10.8.1 (BD) software. The percentage of conjugates, represented by CTV^+^ Emerald^+^ events, was determined based on the gated populations.

### Statistical Analysis.

Statistical analyses were performed using GraphPad Prism v10.3.1. Normality was assessed using the Shapiro–Wilk test. Nonparametric tests (Mann–Whitney, Kruskal–Wallis, and Friedman) were used when data deviated from normality. Proportional comparisons were assessed via a Z-score test for two population proportions (https://www.socscistatistics.com/tests/ztest/). Statistical details are provided in figure legends.

## Supplementary Material

Appendix 01 (PDF)

Movie S1. Live-cell confocal time-lapse of Emerald-LifeAct-expressing MDA-MB-231 cells (green) co-cultured with CMRA-labeled primary NK (pNK) cells (red) in medium containing SYTOX Blue dye (cyan).The right panel shows F-actin rendered in pseudocolor. The sequence captures polarized F-actin remodelling at the immunological synapse during repeated contacts with pNK cells on one side of the target cell. The imaged cells are the same as those depicted in Fig. S1A.

Movie S2. Live-cell confocal time-lapse of Emerald-LifeAct-expressing MDA-MB-231 cells (green) co-cultured with CMRA-labelled primary NK (pNK) cells (red) in medium containing SYTOX Blue (cyan). The right panel shows F-actin rendered in pseudocolour. The sequence shows two target cells: one engages in a brief pNK-cell contact, while the other maintains a sustained ≈1 h contact with continuous F-actin remodelling. The imaged cells are the same as those depicted in Fig. S1B.

Movie S3. Live-cell confocal time-lapse of Emerald-LifeAct-expressing MDA-MB-231 cells (green) co-cultured with CMRA-labelled primary NK (pNK) cells (red) in medium containing SYTOX Blue (cyan). The right panel shows F-actin rendered in pseudocolour. The sequence captures a target cell displaying F-actin remodelling during sequential contacts with three pNK cells. The imaged cells are the same as those depicted in Fig. S1C.

Movie S4. Live-cell confocal time-lapse of Emerald-LifeAct-expressing MDA-MB-231 cells (green) co-cultured with CMRA-labelled primary NK (pNK) cells (red) in medium containing SYTOX Blue (cyan). The right panel shows F-actin rendered in pseudocolour. The sequence shows a target cell that lacks F-actin remodelling at the immunological synapse and undergoes rapid cell death after pNK-cell contact. The imaged cells are the same as those depicted in Fig. S1D.

Movie S5. Live-cell confocal time-lapse of Emerald-LifeAct-expressing MDA-MB-231 cells (green) co-cultured with CMRA-labelled primary NK (pNK) cells (red) in medium containing SYTOX Blue (cyan). The right panel shows F-actin rendered in pseudocolour. The sequence shows a target cell that lacks F-actin remodelling at the immunological synapse and undergoes rapid cell death after pNK-cell contact. The imaged cells are the same as those depicted in Fig. S1E.

Movie S6. Live-cell confocal time-lapse of Emerald-LifeAct-expressing MDA-MB-231 cells (green) co-cultured with CMRA-labelled primary NK (pNK) cells (red) in medium containing SYTOX Blue (cyan). The right panel shows F-actin rendered in pseudocolour. The sequence shows a target cell that lacks F-actin remodelling at the immunological synapse and undergoes rapid cell death after pNK-cell contact. The imaged cells are the same as those depicted in Fig. S1F.

## Data Availability

All study data are included in the article and/or supporting information.
