# Supplementary material for: Cancer cells suppress NK cell activity by actin-driven polarization of inhibitory ligands to the immunological synapse
Source: Proc Natl Acad Sci U S A. 2025 Aug 5;122(32):e2503259122. doi: 10.1073/pnas.2503259122 (PMC12358872; doi:10.1073/pnas.2503259122)
Supplement: Supplementary file 1 — Appendix 01 (PDF) [file pnas.2503259122.sapp.pdf]

PNAS

Supporting Information for
Cancer cells suppress NK cell activity by actin-driven polarization of inhibitory ligands to the immunological synapse

Céline Hoffmann[a,b,1], Liza Filali[a,1], Hannah Wurzer[a,c,1,2], Diogo Pereira Fernandes[a,c], Takouhie Mgrditchian[a], Wanxin Huang[a,c], Flora Moreau[a], Max Krecké[a,c] and Clément Thomas[a,3]

Clément Thomas
Email: clement.thomas@lih.lu

This PDF file includes:

Figures S1 to S11
Legends for Movies S1 to S6

Other supporting materials for this manuscript include the following:

Movies S1 to S6

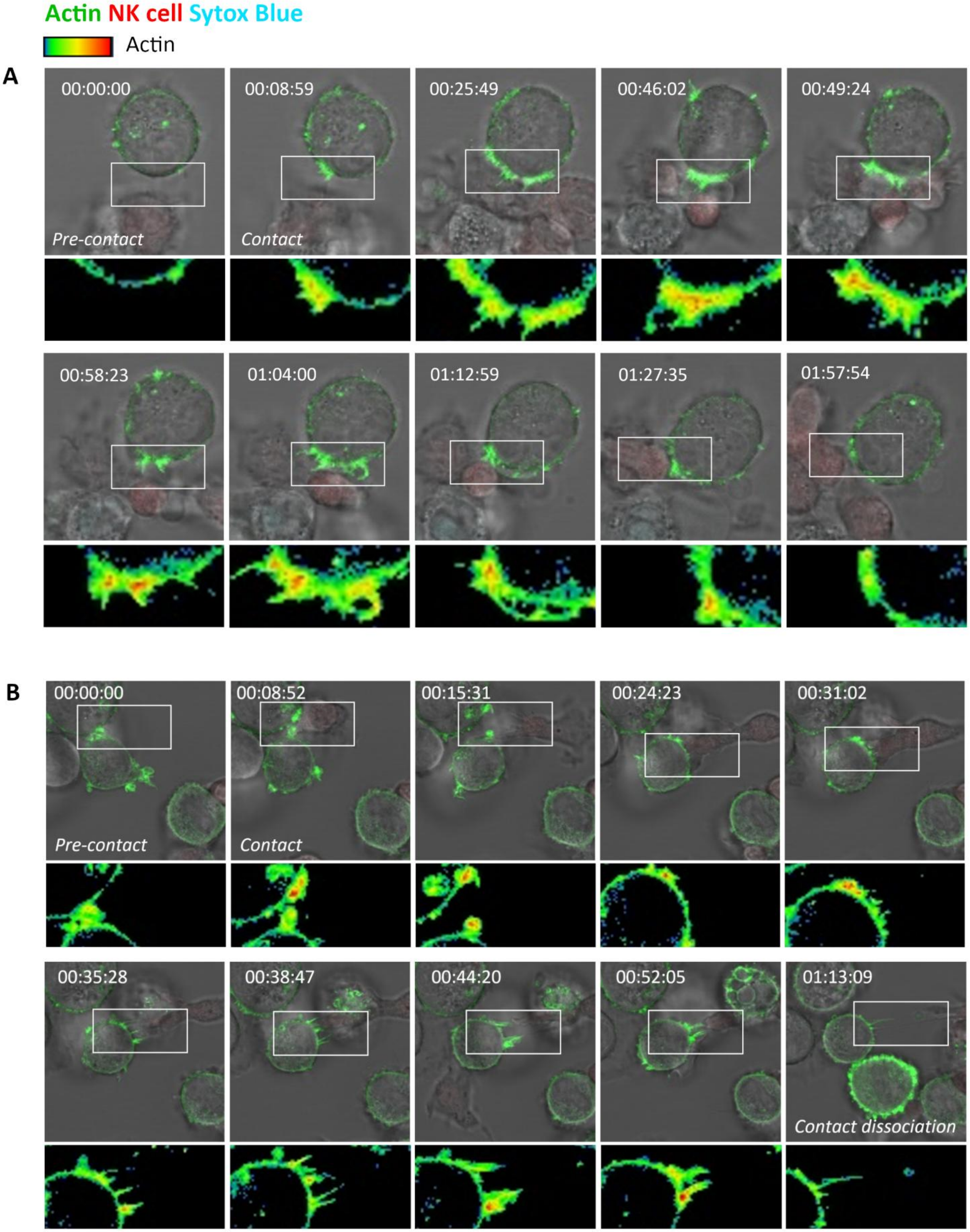
Actin NK cell Sytox Blue
Actin
A
00:00:00
00:08:59
00:25:49
00:46:02
00:49:24
Pre-contact
Contact
00:58:23
01:04:00
01:12:59
01:27:35
01:57:54
B
00:00:00
00:08:52
00:15:31
00:24:23
00:31:02
Pre-contact
Contact
00:35:28
00:38:47
00:44:20
00:52:05
01:13:09
Contact dissociation

C

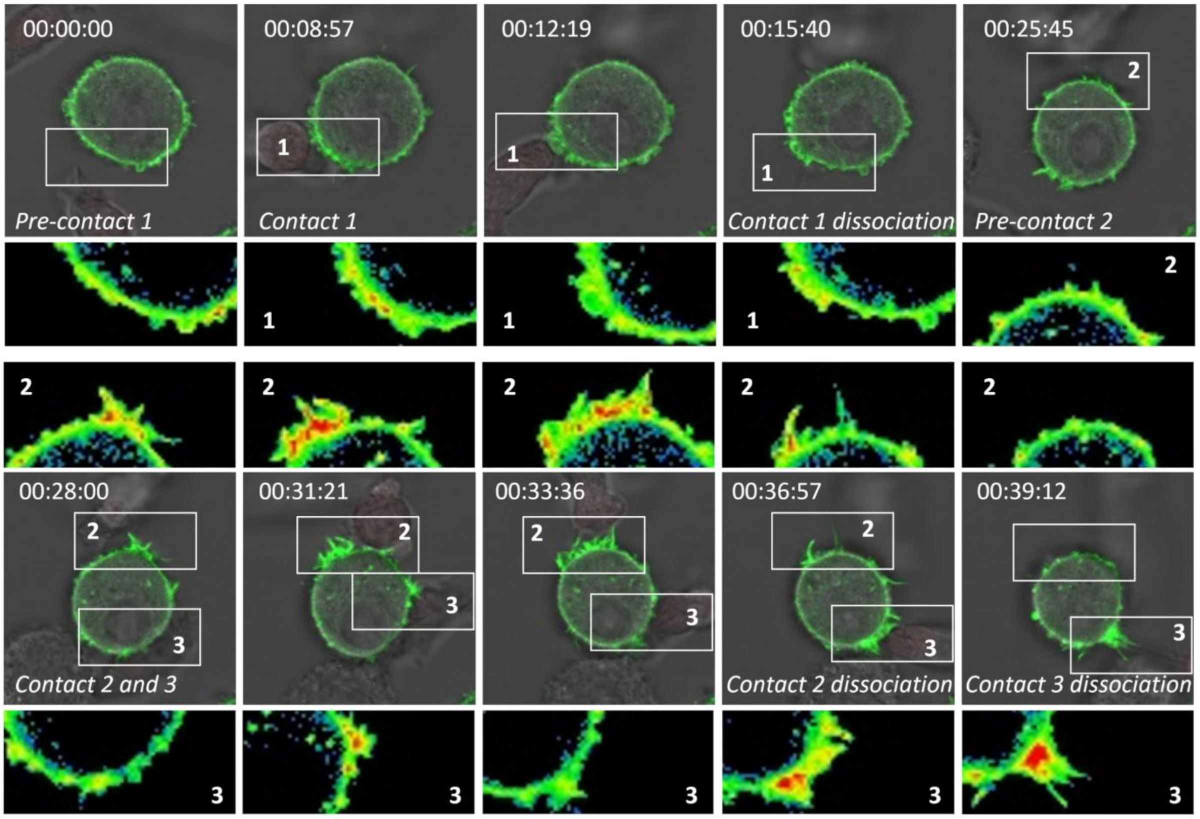
00:00:00
Pre-contact 1
00:08:57
1
Contact 1
00:12:19
1
00:15:40
1
Contact 1 dissociation
00:25:45
2
Pre-contact 2
1
1
1
2
2
2
2
2
2
00:28:00
2
3
Contact 2 and 3
00:31:21
2
3
00:33:36
2
3
00:36:57
2
3
Contact 2 dissociation
00:39:12
3
Contact 3 dissociation
3
3
3
3
3

D

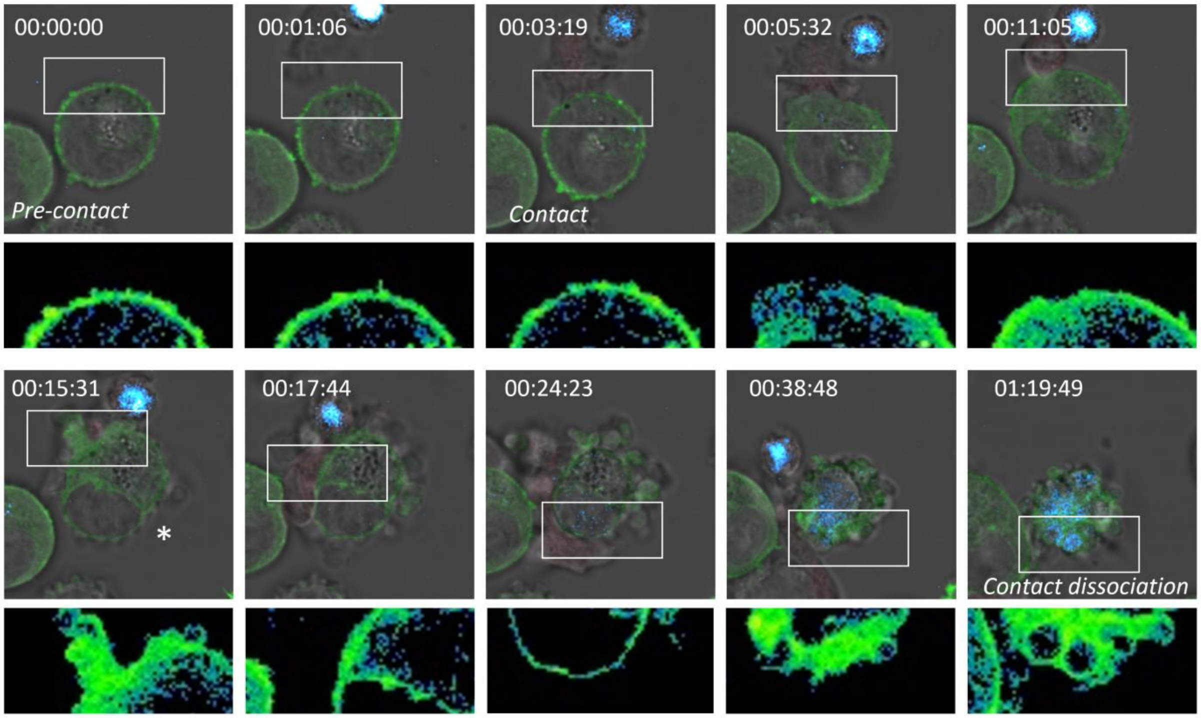
00:00:00
Pre-contact
00:01:06
00:03:19
Contact
00:05:32
00:11:05
00:15:31
*
00:17:44
00:24:23
00:38:48
01:19:49
Contact dissociation

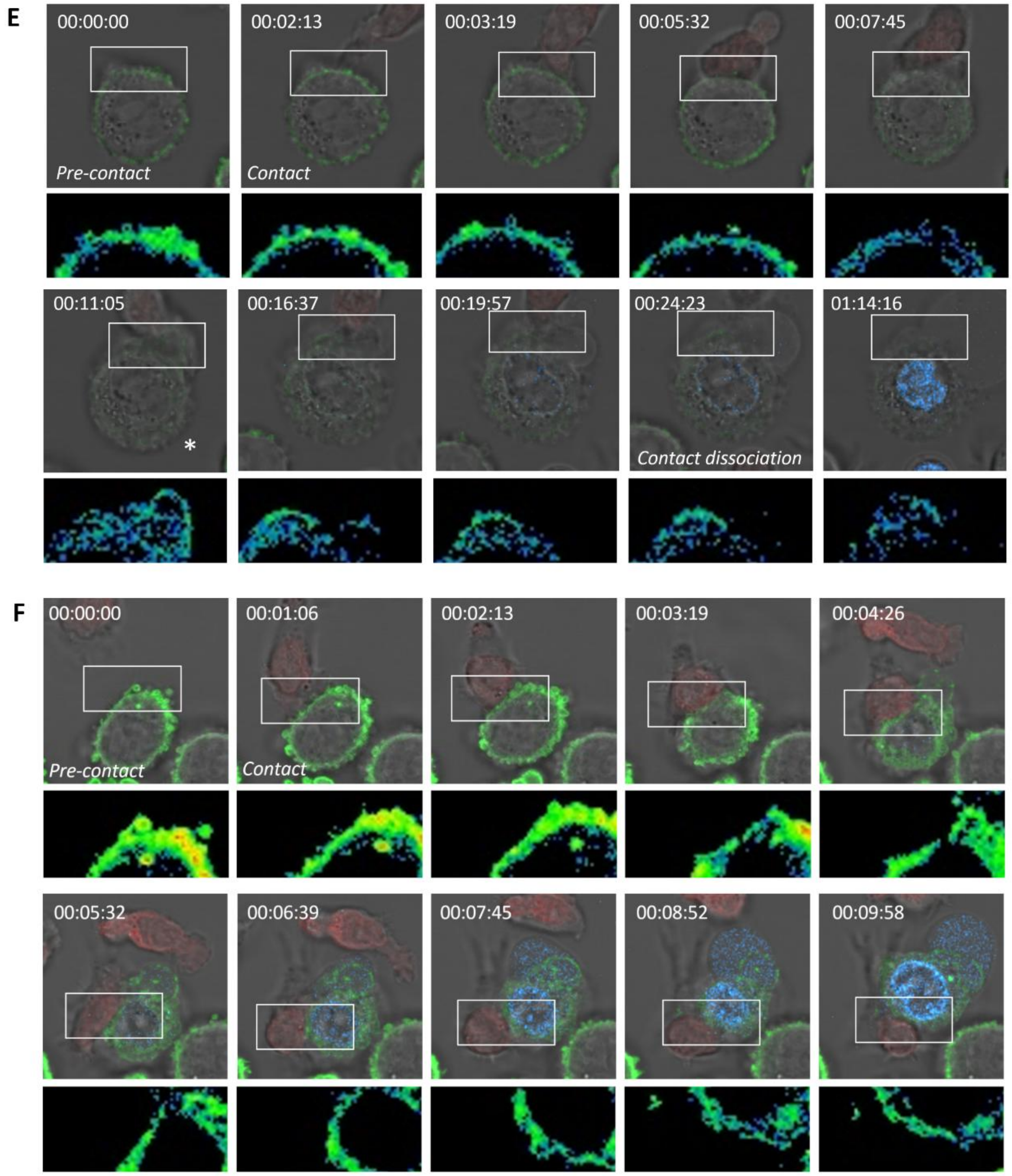

Fig. S1. Live-cell imaging reveals distinct target-cell F-actin configurations associated with differential susceptibility to primary NK-cell-mediated cytotoxicity. Emerald-LifeAct-expressing MDA-MB-231 breast-cancer cells were co-cultured with CMRA-labelled primary NK (pNK) cells in the presence of the viability dye SYTOX Blue. Conjugate formation was monitored by time-lapse confocal microscopy. Panels show representative time-lapse sequences depicting target cell F-actin (green or pseudocolour), pNK cells (red), and SYTOX-positive dead cells (cyan); time 0 marks the pre-contact frame. **(A-C)** Target cells displaying rapid, polarized F-actin remodelling at the immunological synapse following pNK cell contact, associated with no sign of cell death. These panels correspond to Movies S1-S3, respectively. **(D-F)** Target cells showing little or no F-actin remodelling at the immunological synapse, followed by rapid onset of cell death. Asterisks indicate the initiation of membrane blebbing. These panels correspond to Movies S4-S6, respectively.

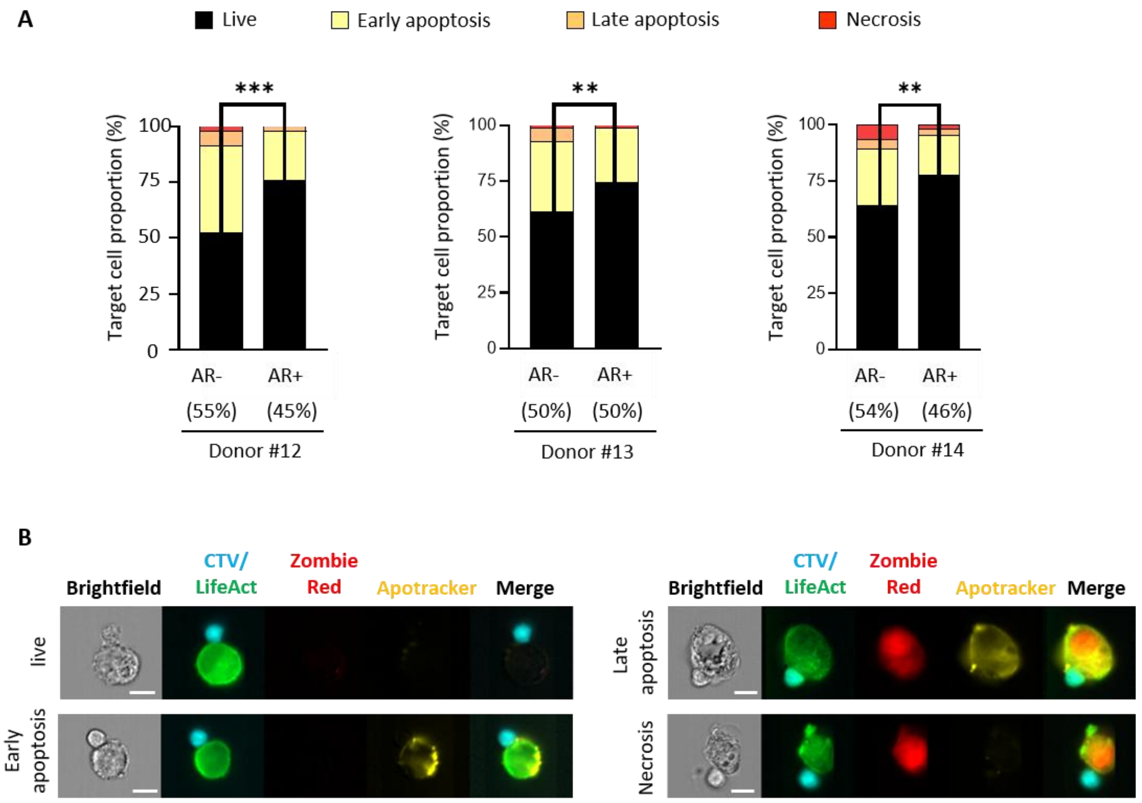

Fig. S2. Synaptic actin remodelling in cancer cells correlates with reduced apoptosis and necrosis. Emerald-LifeAct-expressing MDA-MB-231 cells (green) were incubated with CellTrace Violet (CTV)-labelled primary NK cells (blue) for 60 minutes and stained with Zombie Red and Apotracker to assess apoptosis and necrosis. Experiments were performed wth pNK cells from three healthy donors, analysing at least 140 cell-cell conjugates per condition. Relative Emerald-LifeAct intensity at the immunological synapse was used to classify MDA-MB-231 cells into actin remodeling-positive (AR+, ratio >1) and -negative (AR-, ratio <1) groups. **(A)** The percentage of live (Apotracker-, Zombie Red-), early apoptotic (Apotracker+ Zombie Red-), late apoptotic (Apotracker+ Zombie Red+), and necrotic (Apotracker- Zombie Red+) target cells in conjugates with a pNK cell were quantified by imaging flow cytometry. A Z-score test for two population proportions was used to determine p-values and assess statistical significance between the proportions of live and dying cells. The percentage of AR+ and AR- is indicated for each donor. **(B)** Representative imaging flow cytometry panels of each type of cell conjugate. Scale bars: 10 µm.

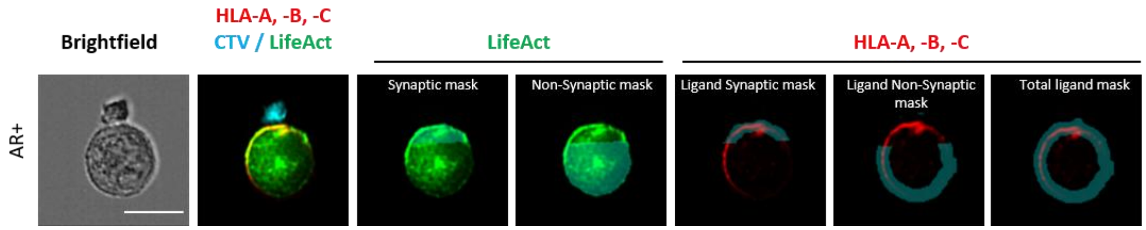

Fig. S3. Description of the masks used for imaging flow cytometry analysis. Emerald-LifeAct-expressing MDA-MB-231 cells (green) were pre-labelled for HLA-A, -B, -C (red) and co-cultured for 40 minutes with CTV-stained primary NK cells (cyan). Representative imaging flow cytometry images of cell-to-cell conjugates between primary NK cells and AR+ MDA-MB-231 cells are presented. A synaptic mask, defined as the proximal third of the cell closest to the synapse, and a non-synaptic mask, encompassing the remaining two-thirds of the cell, were applied. For Emerald-LifeAct intensity measurements, the masks included both the intracellular region and cell membrane. For HLA-A, -B, -C intensity measurements, the masks were restricted to the cell membrane. Relative Emerald-LifeAct and HLA-A, -B, -C, intensities at the IS were calculated as the ratio of mean fluorescence intensity (MFI) in the synaptic mask to the non-synaptic mask. Additionally, a mask covering the entire cell membrane of cell was designed. Masks are shown in light blue. Scale bar: 10 µm.

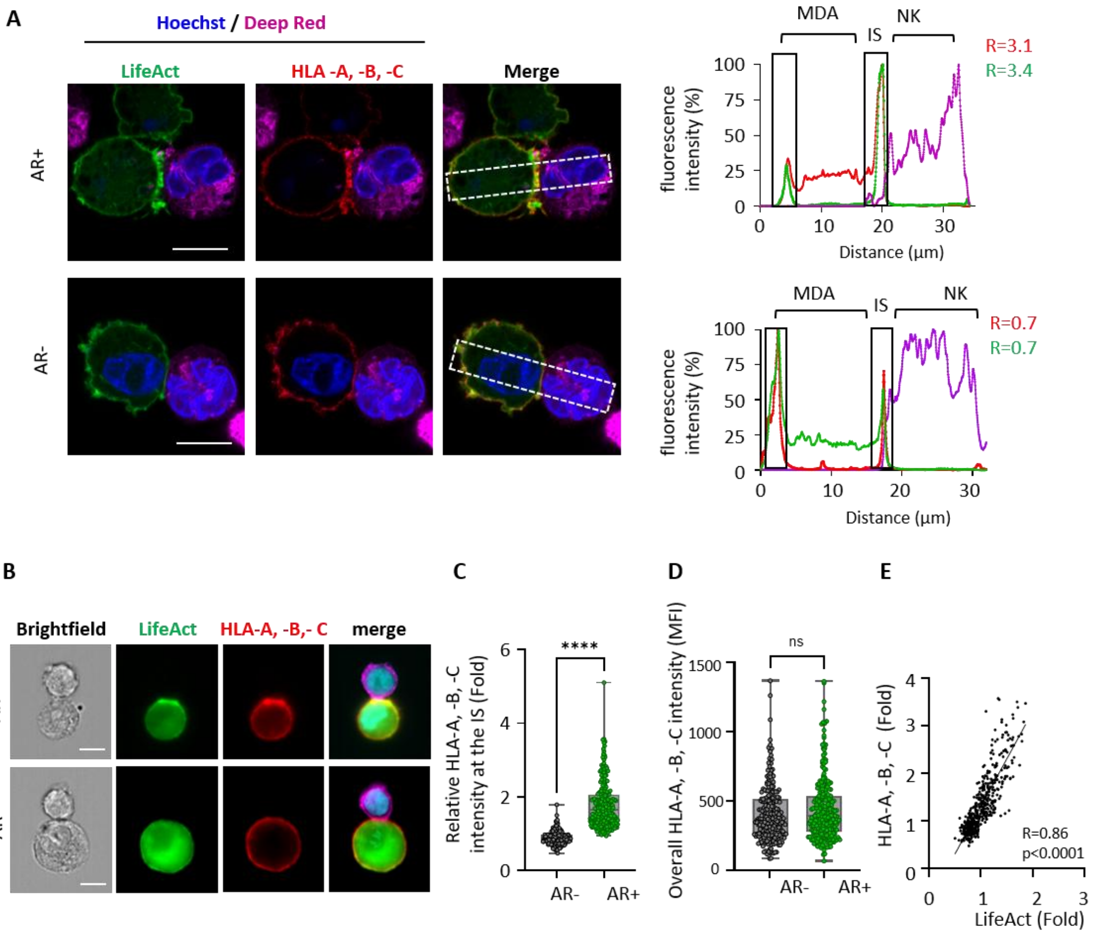

Fig. S4. Polarization of the actin cytoskeleton at the cancer cell side of the immunological synapse correlates with the local accumulation of HL-A, -B, -C molecules during interaction with NK-92MI cells. (A) Emerald-LifeAct-expressing MDA-MB-231 cells (green) were pre-labelled for HLA-A, -B, -C (red) and co-cultured for 40 minutes with Deep-Red-stained NK-92MI cells (magenta), along with Hoechst staining for the nucleus (blue). Representative Airyscan images show cell-to-cell conjugates formed between NK-92MI cells and MDA-MB-231 cells with or without synaptic actin cytoskeleton remodelling at the immunological synapse (AR+ and AR-, respectively). The dashed rectangle indicates the 100-pixel-wide line used to measure mean fluorescence intensity (MFI) for Emerald-LifeAct, HLA-A, -B, -C and Deep Red signals. The upper chart shows MFI profiles for AR+ MDA-MB-231 cells, while the lower graph displays profiles for AR- MDA-MB-231 cells. The relative intensity (R) of HLA-A, -B, -C (red) and Emerald-LifeAct (green) at the synapse are displayed on the plot. **(B-E)** Emerald-LifeAct-expressing MDA-MB-231 cells (green) were pre-labelled for HLA-A, -B, -C (red) and co-cultured for 40 minutes with CD56/Hoechst-stained NK-92MI cells (magenta/cyan). **(B)** Representative imaging flow cytometry images of cell-to-cell conjugates between NK-92MI cells and AR+ or AR- MDA-MB-231 cells. Emerald-LifeAct relative intensity at the synapse was used to classify MDA-MB-231 cells into AR+ and AR- groups (ratio >1 and ratio <1 respectively). Relative HLA-A, -B, -C intensities at the IS in these 2 subgroups are presented **(C)**. The overall MFI of HLA-A, -B, -C across the entire cell membrane of the cancer cell was measured in cell-to-cell conjugates formed between NK-92MI and AR+ or AR- MDA-MB-231 cells **(D)**. Data were collected from 3 independent experiments, with n=250 cell-to-cell conjugates analysed per condition. Statistical significance was determined using the Mann-Whitney test. **(E)** Correlation graph showing the relative Emerald-LifeAct and HLA-A, -B, -C intensities at the IS across the entire population of cell-to-cell conjugates analysed in C-D, without distinguishing between AR+ and AR- MDA-MB-231 cells. The correlation was determined using Spearman's correlation coefficient. Scale bars: 10 µm.

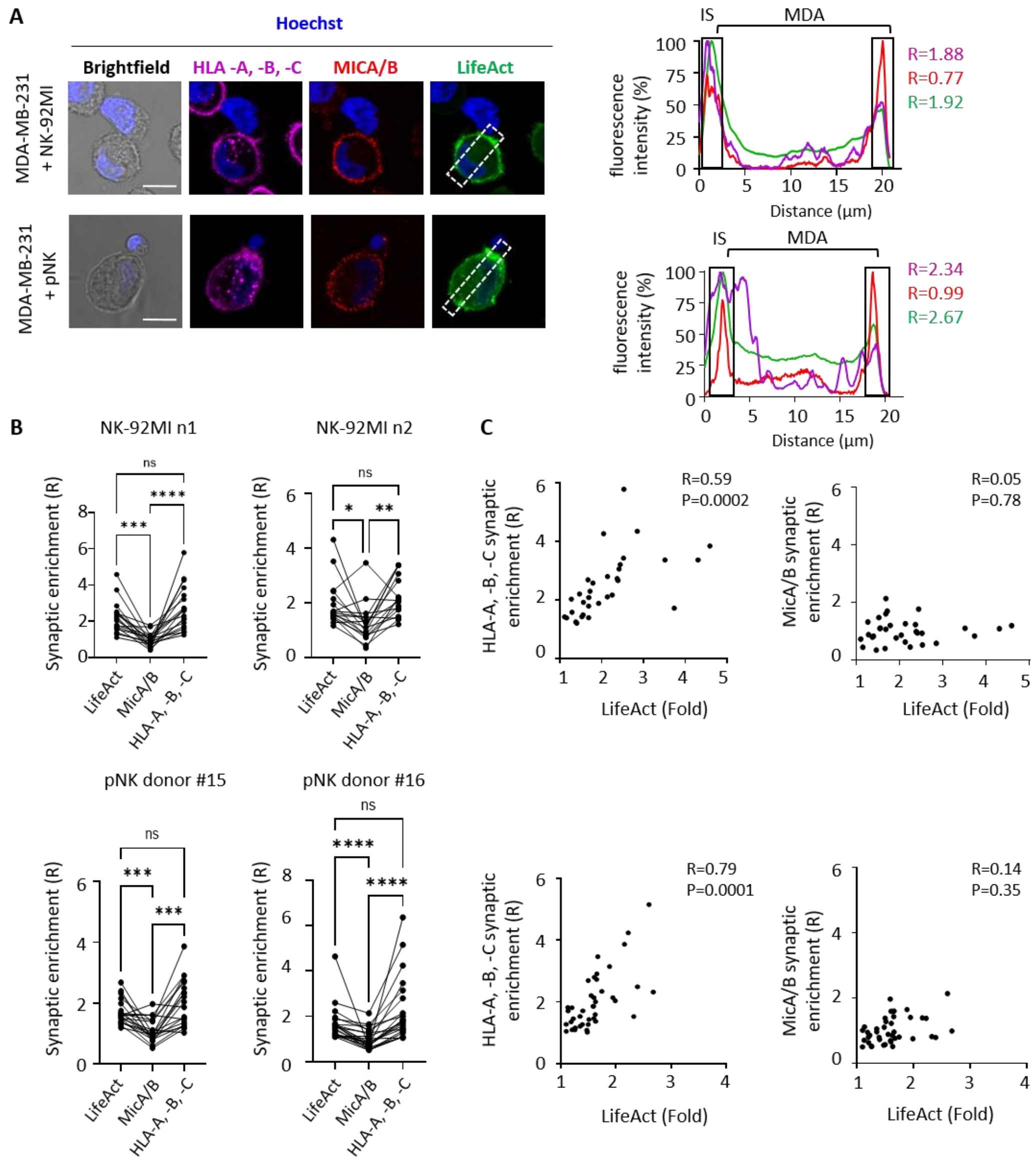

Fig. S5. Polarization of the actin cytoskeleton at the cancer cell side of the immunological synapse does not correlate with strong accumulation of the activating ligands MICA/B during interaction with NK-92MI or primary NK cells. (A) Emerald-LifeAct-expressing MDA-MB-231 cells (green) were pre-labelled for HLA-A, -B, -C (magenta) and MICA/B (red), then co-cultured for 40 minutes with NK-92MI or primary NK (pNK) cells. Nuclei were counterstained with Hoechst (blue). Representative confocal images show NK-cancer cell conjugates in which the actin cytoskeleton is polarized towards the immunological synapse (IS). Images are maximum intensity projections of five-slice Z-stacks. Mean fluorescence intensity of Emerald-LifeAct, HLA-A, -B, -C, and MICA/B in the target cell was measured across 50- or 100-pixel-wide dashed rectangles spanning from the synaptic region to the opposite side of the cell. Corresponding intensity profiles, expressed as a percentage of the maximum fluorescence intensity, are shown in the plots on the right. Synaptic enrichment (R) was calculated as the ratio of maximum intensity at the IS to that at the opposite side of the cell. Scale bars: 10 µm. **(B)** Quantitative comparison of synaptic enrichment for F-actin (LifeAct), MICA/B and HLA-A, -B, -C in 20 NK-92MI-target cell conjugates (upper panels; two independent experiments) and primary NK-target cell conjugates (lower panels; two healthy donors). Statistical significance was assessed by the Friedman test. **(C)** Correlation analysis between synaptic enrichment of Emerald-LifeAct and either HLA-A, -B, -C or MICA/B. Spearman's correlation coefficient (R) and corresponding p values are indicated.

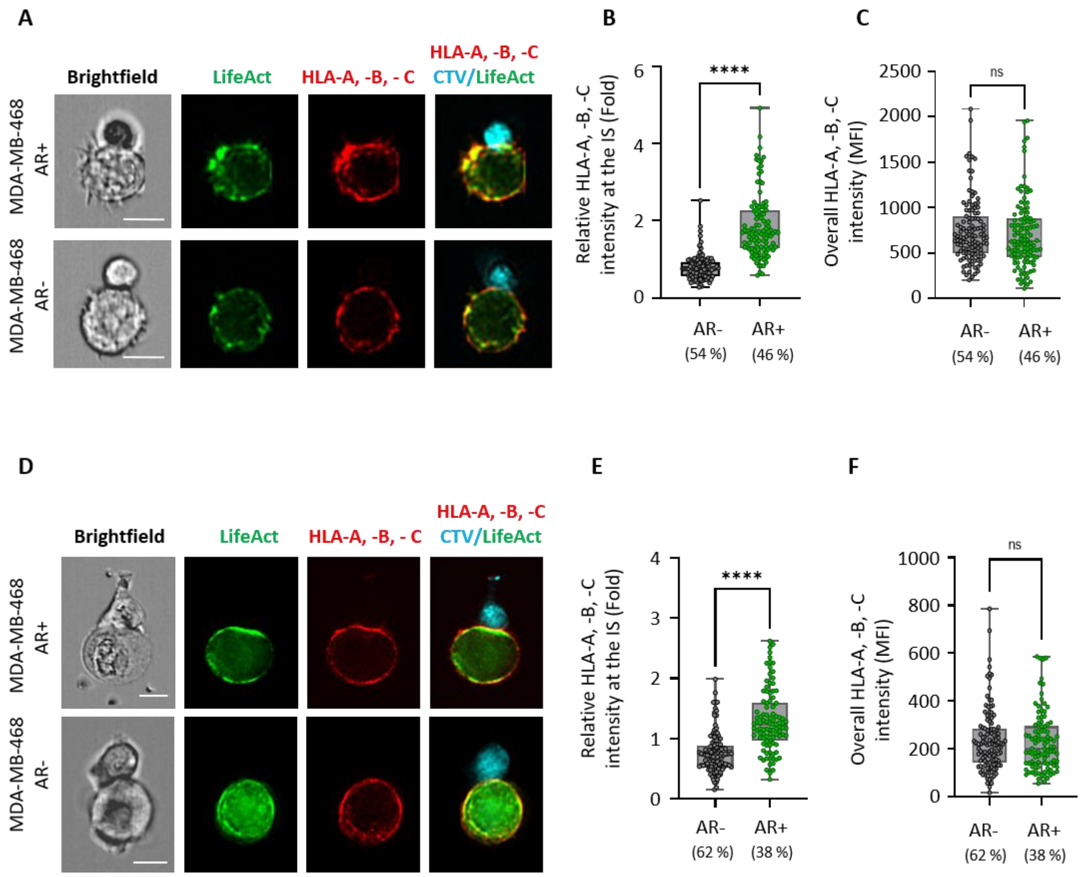

Fig. S6. Association between synaptic polarization of F-actin and HLA-A, -B, -C is conserved in another breast cancer cell line. Emerald-LifeAct-expressing MDA-MB-468 cells (green) were pre-labelled for HLA-A, -B, -C (red) and conjugated with CTV-stained (cyan) primary NK cells **(A-C)** or NK-92MI cells **(D-F)**. **(A and D)** Representative imaging flow cytometry images of cell-to-cell conjugates between NK cells and MDA-MB-468 cells with or without synaptic actin cytoskeleton remodelling at the immunological synapse, (AR+ and AR-, respectively). Scale bars: 10 µm. **(B and E)** Emerald-LifeAct relative intensity at the IS was used to classify MDA-MB-468 cells into AR+ and AR- groups (ratio >1 and ratio <1 respectively). Relative HLA-A, -B, -C intensities at the synapse in these 2 subgroups are presented and the percentage of conjugates in each subgroup is indicated in the axis legend. **(C and F)** The overall mean fluorescence intensity of HLA-A, -B, -C across the entire cell membrane of the cancer cell was measured in cell-to-cell conjugates formed between NK cells and AR+ or AR- MDA-MB-231 cells. **(B and C)** Data were collected from NK cells isolated from 1 donor, with n=100 cell-to-cell conjugates analysed per condition. **(E and F)** Data were collected from 1 experiment with n=100 cell-to-cell conjugates analysed per condition. Statistical significance was determined using a Mann-Witney test.

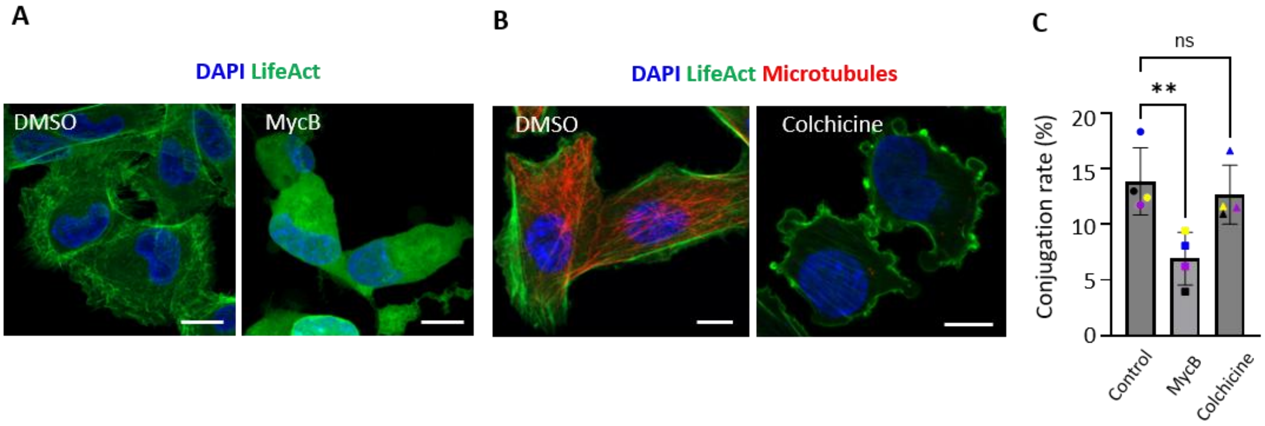

Fig. S7. **Control experiments for drug treatments affecting cytoskeletal organization in cancer cells.** Adherent Emerald-LifeAct-expressing MDA-MB-231 cells (green) were treated with mycalolide B (MycB, an inhibitor of actin filament polymerization) **(A)** or colchicine (a microtubule-disrupting agent) **(B)**. Following treatment, cells were washed, permeabilized and stained for the nucleus (blue) **(A and B)** and microtubules (red) **(B)**. Representative Airyscan images showing MDA-MB-231 cells with control (DMSO) or drug treatment. Maximum intensity projection images generated from 30 (A) or 20 (B) slices of a z-stack are shown. Scale bars: 10 µm. **(C)** Rate of conjugate formation was assessed by flow cytometry. Emerald-LifeAct-expressing MDA-MB-231 cells were treated with MycB or colchicine and conjugated for 40 minutes with NK-92MI at a ratio 1:2 (n=4). Statistical significance was determined using a Kruskal-Wallis test.

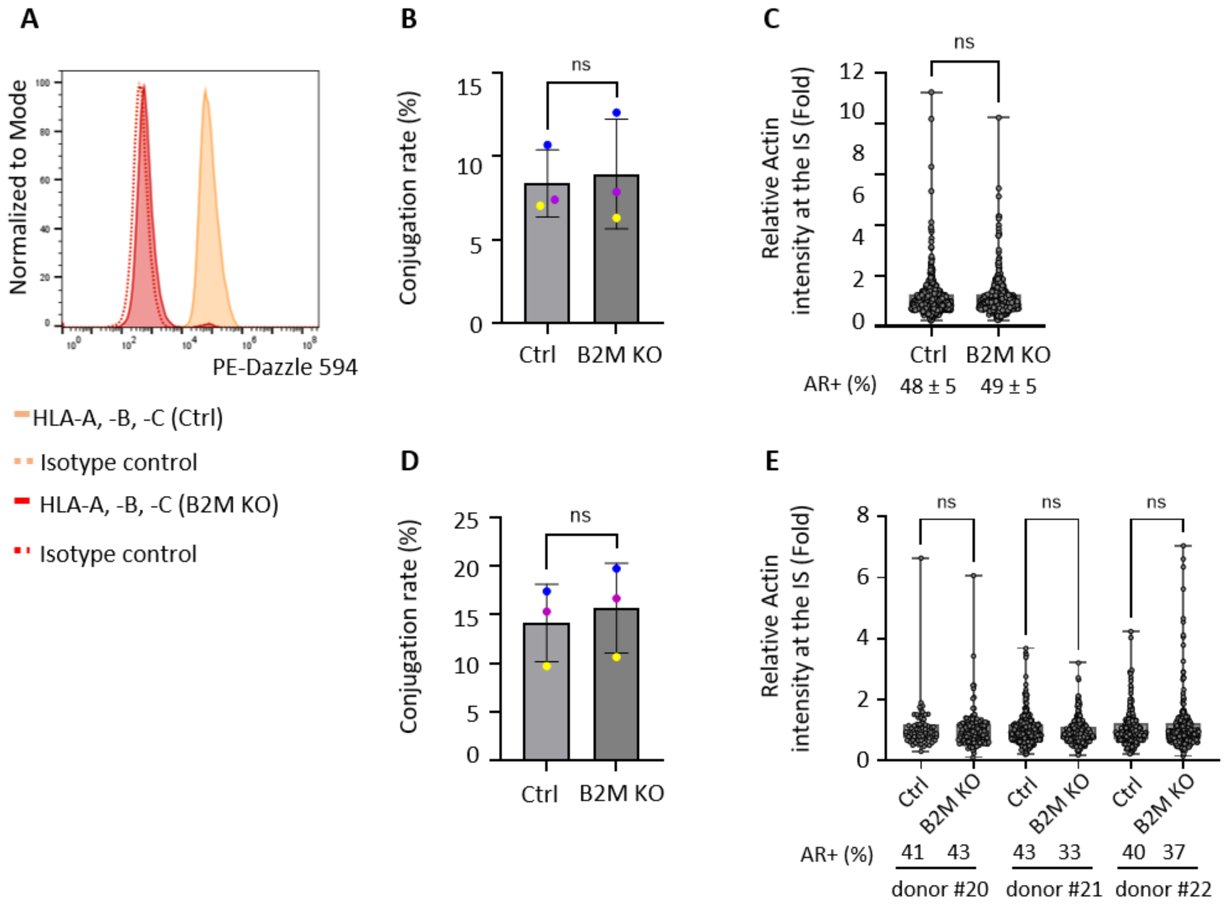

Fig. S8. Analysis of conjugate formation and actin remodeling in B2M KO MDA-MB-231 cells during interaction with NK-92MI or primary NK cells. (A) Flow cytometry analysis of HLA-A, -B, -C surface expression in control and β2-microglobulin knockout (B2M KO) MDA-MB-231 cells. Cells were stained with PE-Dazzle594-conjugated anti-HLA-A, -B, -C antibodies and compared to respective isotype controls. Representative histograms show fluorescence intensity of HLA-A, -B, -C relative to isotype controls in both conditions. **(B and D)** Conjugate formation was assessed by flow cytometry following co-culture of control or B2M KO MDA-MB-231 cells with NK-92MI cells **(B)** or primary NK (pNK) cells **(D)** at a 1:2 target-to-effector ratio for 40 minutes (n=3). **(C and E)** Relative actin enrichment at the immunological synapse was measured by imaging flow cytometry in conjugates formed with NK-92MI **(C)** or pNK cells **(E)**. The percentage of conjugates displaying synaptic actin enrichment in the target cell (AR+, ratio>1) is indicated for each condition. For NK-92MI co-cultures, three independent experiments were performed (n=150 conjugates per experiment). For pNK co-cultures, cells from three healthy donors were used (n>100 conjugates per donor). Statistical significance was assessed using the Mann-Whitney test.

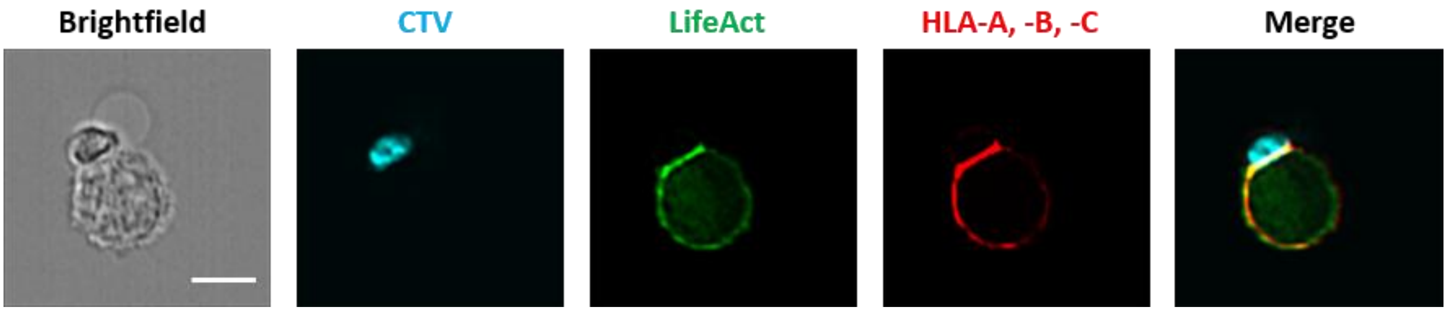

Fig. S9. Control showing that HLA-blocking antibodies reveal a similar synaptic polarization pattern as non-blocking antibodies. Emerald-LifeAct-expressing MDA-MB-231 cells were pre-treated with an HLA-blocking antibody before being conjugated with CTV-labelled primary NK cells for 60 minutes. After conjugation, cells were incubated with a goat anti-mouse Alexa Fluor 555 secondary antibody (red). Scale bar: 10 µm.

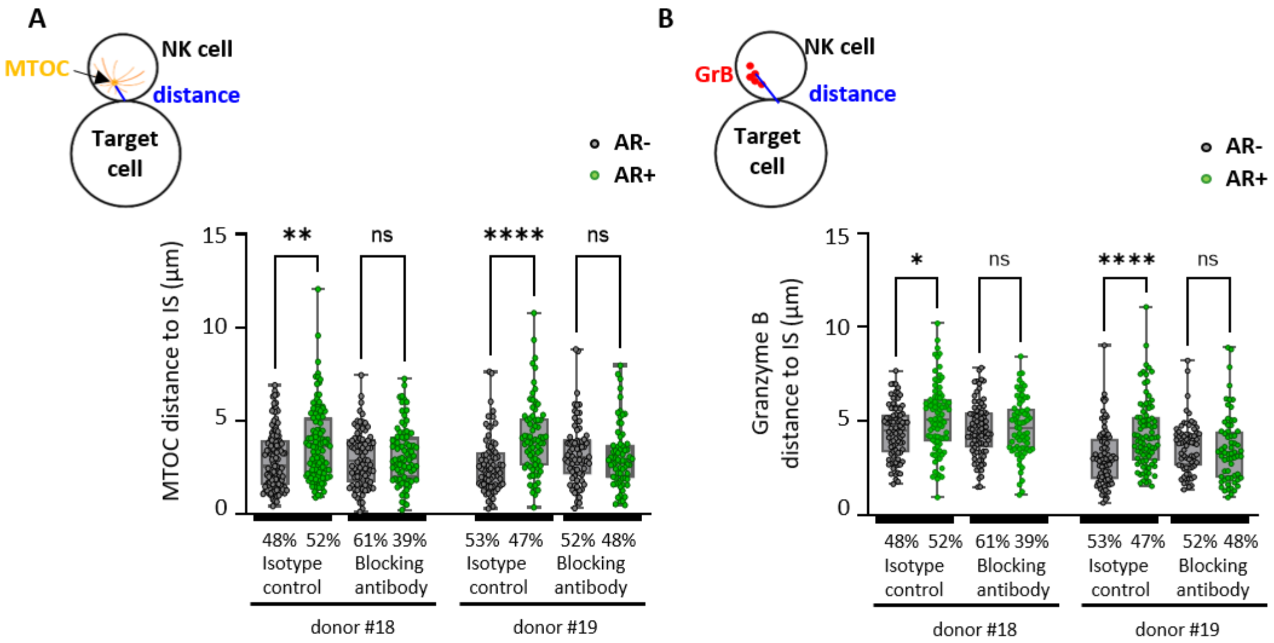

Fig. S10. HLA-blocking antibody counteracts synaptic actin remodelling-driven inhibition of NK cell polarization in another breast cancer cell line. Emerald-LifeAct-expressing MDA-MB-468 cells were pre-treated with either HLA-blocking antibodies or an isotype control antibody before being conjugated with CTV-labelled primary NK cells for 60 minutes. After conjugation, cells were immunolabelled for Granzyme B and γ-Tubulin and analysed using imaging flow cytometry. Emerald-LifeAct relative intensity at the immunological synapse was used to classify MDA-MB-468 cells into AR+ and AR- groups (ratio >1 and ratio <1 respectively). The percentage of conjugates in each subgroup is indicated in the axis legend. NK cell lytic machinery polarization was evaluated by measuring the distance between the MTOC and the IS centre **(A)**, as well as the distance between the Granzyme B centroid and the IS centre **(B)**. Distances for AR+ and AR- subgroups are presented. Data were collected from NK cells isolated from 2 distinct donors, with n=80 cell-to-cell conjugates analysed per condition. Statistical significance was determined using the Mann-Whitney test.

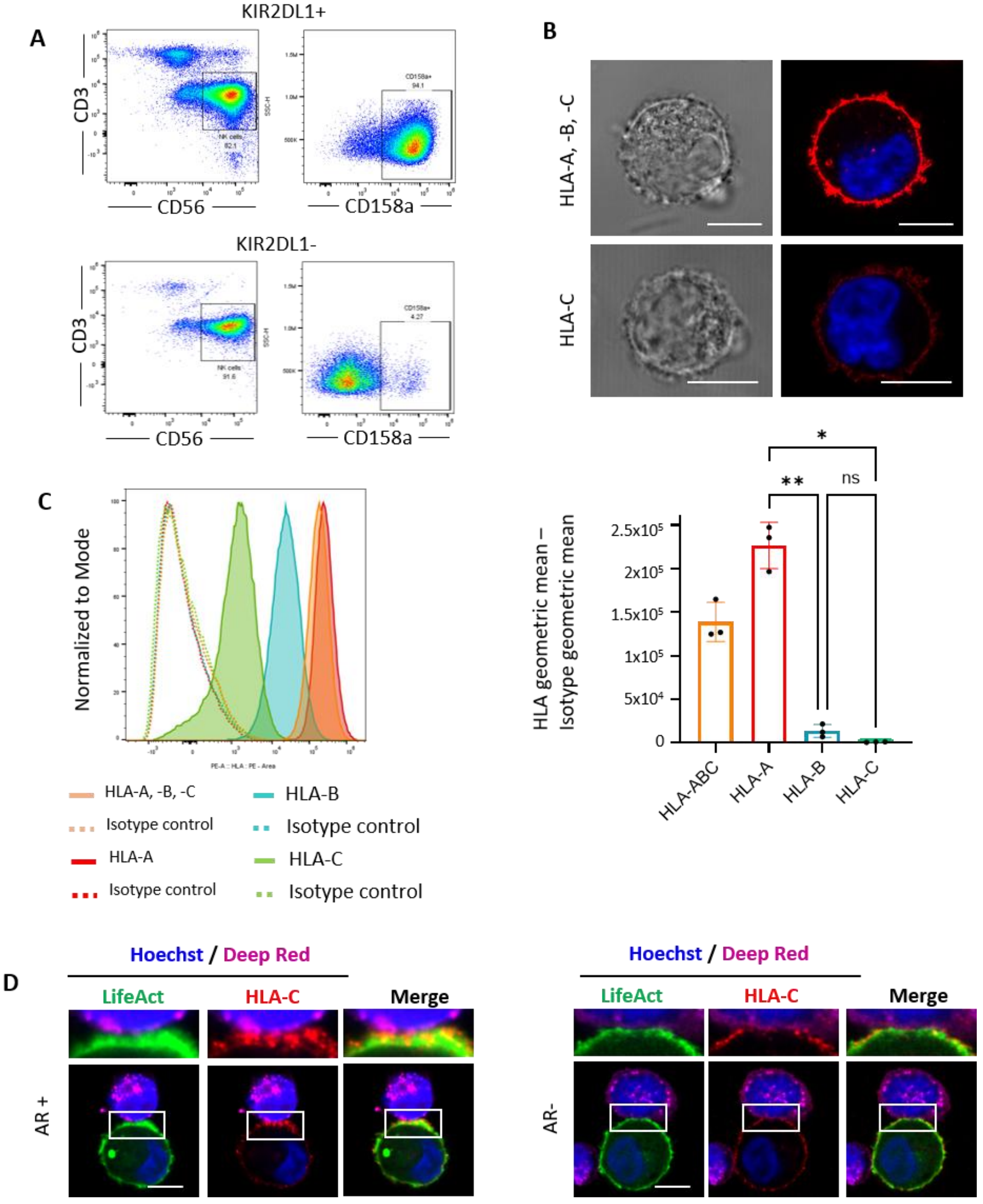

Fig. S11. Characterisation of KIR2DL1+ and KIR2DL1- primary NK cells and HLA-A,-B,-C expression on MDA-MB-231 cells, conjugated or not with primary NK cells. (A) KIR2DL1 expression was analysed by flow cytometry in primary NK cells after sorting and amplification over two weeks prior to experimental use. The first panel shows CD56 and CD3 expression, while the second panel illustrates KIR2DL1 expression in CD56+ CD3- cells. **(B)** MDA-MB-231 cells were pre-labelled with antibodies against HLA-A, -B, -C or HLA-C (red) and stained with Hoechst to visualize nuclei (blue), followed by confocal imaging. **(C)** Flow cytometry analysis of HLA-A, -B, -C expression on MDA-MB-231 cells. MDA-MB-231 cells were stained with PE-conjugated antibodies specific for HLA-A, HLA-B, HLA-C, or pan-HLA-ABC, and compared to respective isotype controls. Representative histograms show fluorescence intensity of each HLA molecule relative to the isotype control. The bar graph displays mean HLA expression levels from three independent experiments. Statistical significance was determined using one-way ANOVA. **(D)** Emerald-LifeAct-expressing MDA-MB-231 cells (green) were pre-labelled for HLA-C (red) and co-cultured for 40 minutes with Deep-Red-stained primary NK cells (magenta) along with Hoechst staining for the nucleus (blue). Representative Airyscan images show cell-to-cell conjugates formed between primary NK cells and MDA-MB-231 cells with or without synaptic actin cytoskeleton remodelling (AR+ and AR-, respectively). Scale bars: 10 µm.

Movie S1 (separate file). Live-cell confocal time-lapse of Emerald-LifeAct-expressing MDA-MB-231 cells (green) co-cultured with CMRA-labeled primary NK (pNK) cells (red) in medium containing SYTOX Blue dye (cyan).The right panel shows F-actin rendered in pseudocolor. The sequence captures polarized F-actin remodelling at the immunological synapse during repeated contacts with pNK cells on one side of the target cell. The imaged cells are the same as those depicted in Fig. S1A.

Movie S2 (separate file). Live-cell confocal time-lapse of Emerald-LifeAct-expressing MDA-MB-231 cells (green) co-cultured with CMRA-labelled primary NK (pNK) cells (red) in medium containing SYTOX Blue (cyan). The right panel shows F-actin rendered in pseudocolour. The sequence shows two target cells: one engages in a brief pNK-cell contact, while the other maintains a sustained ≈1 h contact with continuous F-actin remodelling. The imaged cells are the same as those depicted in Fig. S1B.

Movie S3 (separate file). Live-cell confocal time-lapse of Emerald-LifeAct-expressing MDA-MB-231 cells (green) co-cultured with CMRA-labelled primary NK (pNK) cells (red) in medium containing SYTOX Blue (cyan). The right panel shows F-actin rendered in pseudocolour. The sequence captures a target cell displaying F-actin remodelling during sequential contacts with three pNK cells. The imaged cells are the same as those depicted in Fig. S1C.

Movie S4 (separate file). Live-cell confocal time-lapse of Emerald-LifeAct-expressing MDA-MB-231 cells (green) co-cultured with CMRA-labelled primary NK (pNK) cells (red) in medium containing SYTOX Blue (cyan). The right panel shows F-actin rendered in pseudocolour. The sequence shows a target cell that lacks F-actin remodelling at the immunological synapse and undergoes rapid cell death after pNK-cell contact. The imaged cells are the same as those depicted in Fig. S1D.

Movie S5 (separate file). Live-cell confocal time-lapse of Emerald-LifeAct-expressing MDA-MB-231 cells (green) co-cultured with CMRA-labelled primary NK (pNK) cells (red) in medium containing SYTOX Blue (cyan). The right panel shows F-actin rendered in pseudocolour. The sequence shows a target cell that lacks F-actin remodelling at the immunological synapse and undergoes rapid cell death after pNK-cell contact. The imaged cells are the same as those depicted in Fig. S1E.

Movie S6 (separate file). Live-cell confocal time-lapse of Emerald-LifeAct-expressing MDA-MB-231 cells (green) co-cultured with CMRA-labelled primary NK (pNK) cells (red) in medium containing SYTOX Blue (cyan). The right panel shows F-actin rendered in pseudocolour. The sequence shows a target cell that lacks F-actin remodelling at the immunological synapse and undergoes rapid cell death after pNK-cell contact. The imaged cells are the same as those depicted in Fig. S1F.
